# GATOR1 signaling defects promote astrocytic metabolic rewiring and excitatory neurotransmitter cycling

**DOI:** 10.1038/s44319-026-00846-w

**Published:** 2026-07-03

**Authors:** Imane Hadj-Aissa, Maéline Muller, Jorge Soliz, Chantelle F Sephton, Paul A Dutchak

**Affiliations:** 1 Department of Psychiatry and Neuroscience, CERVO Brain Research Centre, Université Laval, Québec City, QC G1J 2G3 Canada; 2 Centre de Recherche de l’Institute Universitaire de Cardiologie et de Pneumologie de Québec, Université Laval, Québec City, QC G1V 4G5 Canada

**Keywords:** Metabolism, Neuroscience

## Abstract

GATOR1 is an evolutionarily-conserved negative regulator of mTORC1-dependent signal transduction with pathogenic mutations linked to epilepsy, infantile spasms, and autism spectrum disorders. While a biochemical role of GATOR1 in amino acid-signaling is established, its cell-type specific contributions within the brain remain poorly defined. Here, we show that loss of GATOR1 function in astrocytic cells disrupts mitochondrial metabolism, with a selective dysfunction of the electron transport chain Complex II leading to elevated reactive oxygen species (ROS) and redox imbalance. These changes are accompanied by compensatory increases in antioxidant regulatory systems including superoxide dismutase, but remain insufficient to ameliorate the increased ROS. GATOR1-deficient astrocytes show metabolic rewiring marked by enhanced expression of glutamate uptake and glutamine synthesis pathways that contribute to the glutamate-glutamine cycle governing neuronal glutamine availability and synaptic homeostasis. In vivo, GATOR1 deficiency results in progressive astrocytic reactivity, seizures, and a reduced lifespan. These findings demonstrate that GATOR1 function is critical to coordinate astrocytic mitochondrial activity and neurotransmitter cycling pathways, establishing a novel link between intracellular amino acid-signaling in astrocytes and excitatory neural network homeostasis.

## Synopsis

Defective GATOR1 signaling in astrocytes induces mTORC1-dependent metabolic rewiring that enhances glutamine production and reshapes neuron-glia metabolic coupling. These alterations promote excitatory network imbalance and seizures in vivo.

- GATOR1-deficient astrocytes undergo metabolic rewiring, characterized by altered mitochondrial function, redox imbalance, and selective impairment in Complex II-dependent respiration.
- Metabolic reprograming enhances glutamine synthesis and release through upregulation of glutamine synthetase and associated transport pathways, reshaping neuron-glia metabolic coupling.
- Astrocyte-derived metabolic changes are sufficient to alter neuronal excitatory programs, increasing glutamine utilization and vesicular glutamate loading in neurons, which contribute to increased seizure sensitivity.

## Abstract (web summary)

Defective GATOR1 signaling in astrocytes induces mTORC1-dependent metabolic rewiring that enhances glutamine production and reshapes neuron-glia metabolic coupling. These alterations promote excitatory network imbalance and seizures in vivo.

## Graphical abstract

**Figure:**
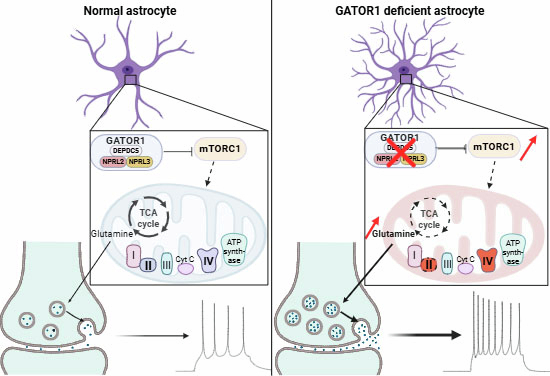

**Figure:**
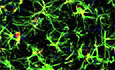

## Introduction

The metabolic coupling of distinct cell populations in the central nervous system (CNS) is a tightly coordinated and essential process for the development and maintenance of neuronal activity and brain function (Bonvento and Bolanos, 2021; Mann et al, 2021). The evolutionarily-conserved protein complex called GAP activity toward Rag1 (GATOR1) functions as a key regulator of intracellular amino acid homeostasis, coordinating cellular growth and mRNA translation in response to nutrient availability (Bar**-**Peled et al, 2013; Dokudovskaya and Rout, 2015; Neklesa and Davis, 2009). Somatic and mosaic mutations in GATOR1 subunits have been identified in patients with multiple forms of epilepsy, including familial focal epilepsy with variable foci (FFEVF), sleep-related nocturnal seizures (NFLE), temporal lobe epilepsy (TLE), and infantile spasms; in addition to some cases of autism spectrum disorders (ASD) (Baldassari et al, 2019; Ishida et al, 2013; Picard et al, 2014; Ricos et al, 2016). Single-cell transcriptomic studies have found that these mutations can occur in both neurons and astrocytes, suggesting that disease progression may arise through distinct cellular mechanisms involving different cell types (Baldassari et al, 2025). Although neuronal GATOR1 knockout models consistently develop spontaneous seizures and die prematurely (Dentel et al, 2022; Hui et al, 2022; Ishida et al, 2022), the impact of GATOR1 dysfunction in the astrocytic population and its impact on neural network excitability remains unclear.

The GATOR1 complex is expressed from three requisite genes called *Nprl2* (nitrogen permease regulator-like 2), *Nprl3*, and *Depdc5* (DEP domain containing 5), and functions as a GTPase-activating protein complex (GAP) for RAG (Ras-related GTP-binding protein) GTPases found on the lysosomal surface (Bar**-**Peled et al, 2013). During conditions of low intracellular amino acids or starvation, GATOR1 activity inhibits mTORC1 (mammalian target of rapamycin complex 1) activity to downregulate anabolic processes and protein synthesis, conserving cellular amino acids and energy. In the presence of amino acids, GATOR1 activity is repressed by inhibitory interactions with a distinct protein complex called GATOR2, which is further regulated through upstream protein interactions, including SESTRIN2 and CASTOR1/2 in response to leucine and arginine abundance, respectively (Chantranupong et al, 2016; Wolfson et al, 2016). These regulatory interactions position GATOR1 as a central mediator of amino acid-dependent control of mTORC1, establishing a conserved link between nutrient availability and metabolic stress responses.

Within cells, excessive mTORC1 activity can drive metabolic rewiring to support the high energy and biosynthetic demands of growth and proliferation, a hallmark of many cancers (Laplante and Sabatini, 2012; Panwar et al, 2023; Saxton and Sabatini, 2017). In the brain however, astrocytes contribute to the basal metabolic environment within the tissue, providing essential substrates that sustain neuronal activity and synaptic functions (Batiuk et al, 2020; Bonvento and Bolanos, 2021). Accordingly, astrocytes can exhibit pronounced metabolic plasticity to meet local energy demands (Zhang et al, 2023) and mitigate oxidative stress through redox-regulating pathways (Vicente-Gutierrez et al, 2019). Astrocytes also mediate neurotransmitter homeostasis, particularly via the glutamate-glutamine cycle, which couples their metabolism to neuronal synaptic activity (Tani et al, 2014). Despite growing insights into the cell-autonomous functions of GATOR1-mediated amino acid sensing, the role of GATOR1 in mediating astrocyte-neuronal metabolic coupling is not well understood. These considerations raise the question of whether GATOR1 can impact astrocytic metabolism in ways that can directly affect neuronal activity.

Here, we show that loss of GATOR1 function in astrocyte-derived C8-D1A cells impairs mitochondrial metabolism and electron transport chain activity, leading to elevated levels of reactive oxygen species. In response, GATOR1-defective cells undergo metabolic rewiring that enhances glutamine synthesis and secretion, thereby providing the necessary substrates used for excitatory glutamatergic signaling in neurons. Consistent with these mechanistic findings, CRE-mediated deletion of GATOR1 function results in progressive astrocytic reactivity, seizures, and premature death. Collectively, these results support that GATOR1-dependent signaling plays a critical role in regulating the metabolic coupling of astrocytes and neurons, linking intracellular amino acid-signaling pathways to seizure susceptibility.

## Results and discussion

### GATOR1 dysfunction increases mTORC1 activity and age-dependent reactive astrocytosis

**Figure 1:**
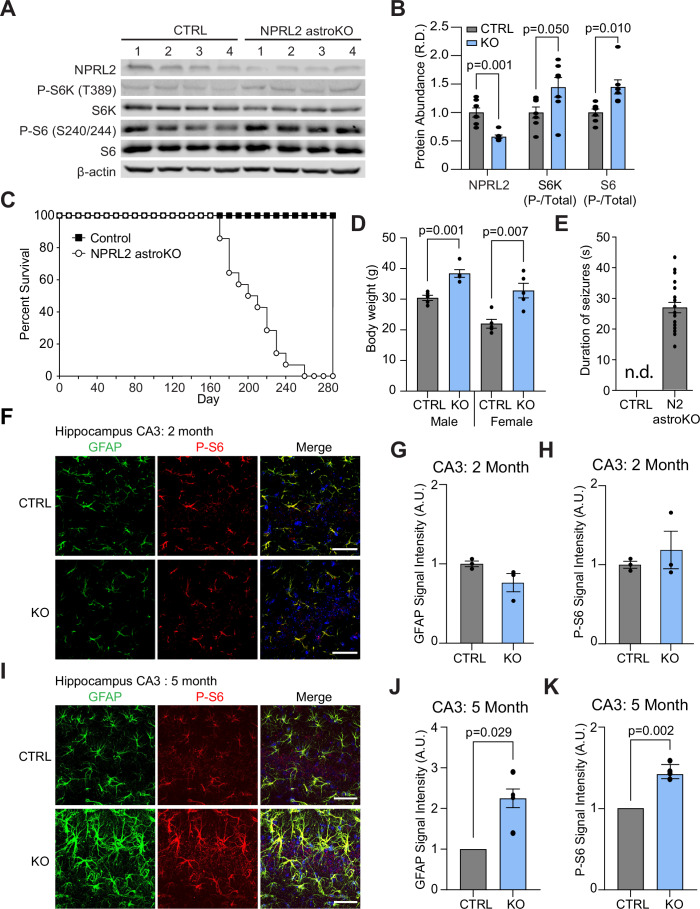
GATOR1 defects induce astrocytic reactivity and seizures. (**A**) Representative western blot analysis from one independent experiment of NPRL2, P-S6K (T389), S6K, P-S6 (S240/244), S6 and β-actin in brain protein extracts from NPRL2 astroKO and control (CTRL) mice at 5 months of age (*n* = 4 mice per genotype, independent biological replicates). (**B**) Quantification of protein abundance. Relative density (R.D.) of P-S6K (T389) was normalized to total S6K and β-actin, P-S6 (S240/244) to total S6 and β-actin, and NPRL2 to β-actin (*n* = 7 mice per genotype pooled across independent experiments; biological replicates). (**C**) Kaplan–Meier survival analysis of NPRL2 astroKO and CTRL mice (*n* = 14 mice per genotype). (**D**) Body mass of male and female NPRL2 astroKO and CTRL mice at 5 months (*n* = 5 mice per genotype). (**E**) Average seizure duration in 5-month-old NPRL2 astroKO and CTRL mice recorded over a continuous 48-h period (*n* = 3 mice per genotype; n.d. = not detected). (**F**) Representative confocal microscopy images of hippocampal CA3 sections stained for GFAP, P-S6 and DAPI in 2-month old mice. Scale bar, 40 µm. (**G**,** H**) Quantification of average (**G**) GFAP and (**H**) P-S6 signal intensity (AU, arbitrary units) in the CA3 region of 2-month-old mice (*n* = 3 mice per genotype; biological replicates). (**I**) Representative confocal microscopy images of hippocampal CA3 sections stained for GFAP, P-S6 and DAPI in 5-month-old mice. Scale bar, 40 µm. (**J**,** K**) Quantification of average (**J**) GFAP and (**K**) P-S6 signal intensity in the CA3 region of 5-month-old mice (*n* = 4 independent biological experiments; values represent KO normalized to matched WT within each experiment; two-tailed one-sample *t-*test vs 1 A.U., arbitrary units). Data were shown as the mean ± S.E.M. Two-tailed Welch’s *t*-test was used for two-group comparisons unless otherwise indicated. Source data are available online for this figure.

**Figure EV1:**
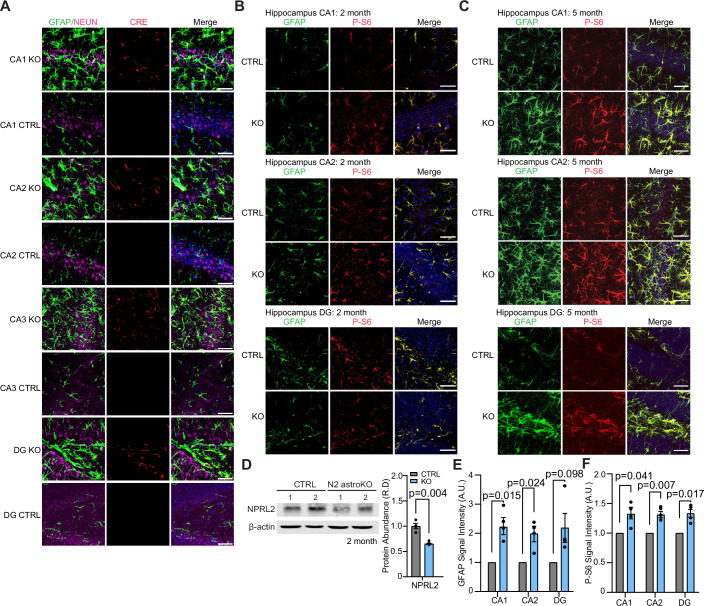
GATOR1 deficiency sensitizes mice to age-dependent reactive astrocytosis. (**A**) Representative confocal microscopy images of hippocampal CA1, CA2, CA3, and dentate gyrus (DG) sections at 5-month-old NPRL2 astroKO and control (CTRL) mice stained for GFAP, NEUN, CRE and DAPI. Scale bars = 40 µm. (**B**,** C**) Representative confocal microscopy images of hippocampal CA1, CA2, and DG regions from NPRL2 astroKO and control mice at (**B**) 2 months and (**C**) 5 months of age, stained for GFAP, P-S6 and DAPI. Scale bars = 40 µm. (**D**) Representative western blot analysis of NPRL2 and β-actin from NPRL2 astroKO and control brain protein extracts at 2 months of age. Quantification of NPRL2 abundance relative to β-actin is shown as relative density (R.D.) normalized to β-actin and includes all animals analyzed across two independent biological experiments (*n* = 4 mice per genotype). Welch’s *t*-test was used for comparisons. (**E**,** F**) Quantification of average (**E**) GFAP and (**F**) P-S6 signal intensity in hippocampal CA1, CA2, and DG regions of control and NPRL2 astroKO mice at 5 months of age (*n* = 4 independent biological experiments; values represent KO normalized to matched WT within each experiment; two-tailed one-sample *t-*test vs 1 AU, arbitrary units). Data were shown as the mean ± SEM.

GATOR1 defects in neuronal populations cause early-onset seizures and lethality (Hui et al, 2022; Ishida et al, 2022; Yuskaitis et al, 2018), but its function in astrocytes is not clear. To investigate the contribution of GATOR1 in astrocytes, we generated an NPRL2 astrocytic knockout model by crossing *Nprl2*-floxed males with *Gfap-ires-*Cre females, to generate *Nprl2*^*loxP/loxP*^*; Gfap-ires-*Cre^*+/WT*^ (NPRL2 astroKO) offspring (Dutchak et al, 2015; Gregorian et al, 2009). PCR was used to confirm genotypes and western blot analysis verified NPRL2 reduction within the brain tissue (Fig. 1A,B). As validated NPRL2 antibodies for immunohistochemistry (IHC) are not available, we used CRE expression to confirm its presence within the GFAP-positive astrocytes (Fig. EV1A). Western blot analysis of brain lysates from NPRL2 astroKO mice showed a modest, but statistically significant increase in mTORC1 signaling, with increased phosphorylation of S6 kinase (S6K) and S6 compared to controls (Fig. 1A,B). These data were consistent with a loss of amino acid-dependent regulation of mTORC1 activity, similar to enhanced mTORC1 signaling caused by TSC defects (Uhlmann et al, 2002).

During routine colony maintenance, we observed that NPRL2 astroKO animals displayed sudden mortality beginning at 5 months of age, with no animals surviving beyond 8.5 months (Fig. 1C). Both male and female NPRL2 astroKO mice showed a significant increase in body mass relative to age-matched controls (Fig. 1D); in contrast to neuronal NPRL2 or NPRL3 KO mice, which show reduced body mass and die within the first postnatal month (Hui et al, 2022; Ishida et al, 2022). Although not directly examined, these data raise the possibility of altered hypothalamic metabolic regulation. Video recordings of the animals revealed multiple daily seizures (~30 s) at 5 months in NPRL2 astroKO mice, whereas littermate controls remained seizure-free (Movie EV1; Fig. 1E).

To determine whether seizure activity was accompanied by astrocytic reactivity, a process implicated in seizure generation (Robel et al, 2015; Zhang et al, 2023), we performed IHC and confocal microscopy on brain sections collected either before (2 months) or after (5 months) seizure onset. At 2 months, neither astrocytic morphology nor mTORC1 signaling differed between genotypes (Figs. 1F–H and EV1B), despite the similar reduction of NPRL2 expression across ages (Figs. 1A and EV1D). By 5 months, NPRL2 astroKO mice showed pronounced GFAP-positive morphological changes characteristic of astrocytic reactivity and increased P-S6 staining across all hippocampal regions (Figs. 1I–K and EV1C,E,F). Although certain GFAP-*Cre* alleles have been reported to drive recombination in a small subset of neurons from GFAP-expressing progenitors within neurogenic zones (e.g., subventricular and subgranular zones), the extent to which these contribute to seizures remains uncertain, and the observed cellular changes are consistent with a reactive astrocytic phenotype (Malatesta et al, 2003; Robel et al, 2015). The delayed onset of the seizures remains unclear, but suggests that age-related metabolic shifts may contribute (Ding et al, 2021; Matias et al, 2023; Shen et al, 2024). Collectively, these data show that GATOR1 is necessary to restrict astrocytic reactivity and prevent the onset of seizures in vivo.

### Metabolic reprogramming of GATOR1-deficient astrocytes

**Figure EV2:**
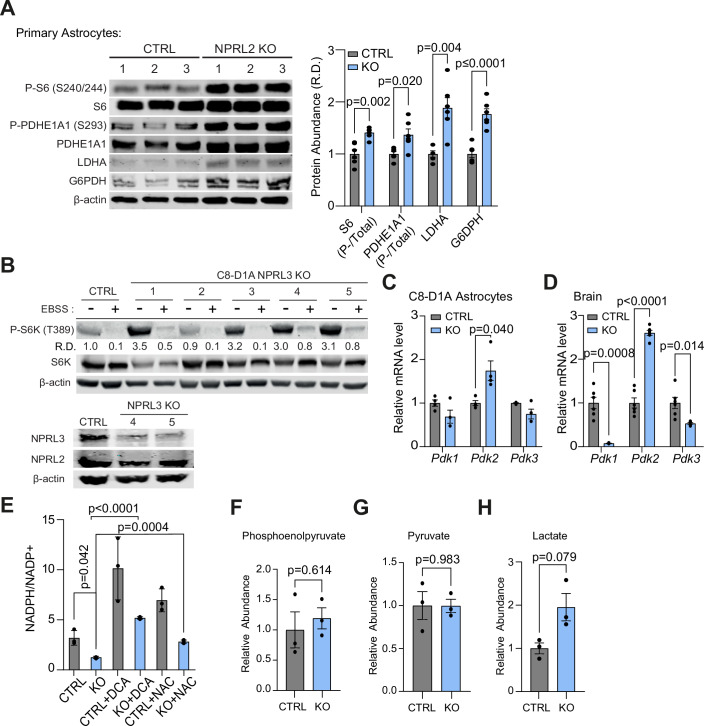
Glycolysis and TCA cycle metabolic changes in GATOR1-deficient cells. (**A**) Western blot analysis of P-S6 (S240/244), S6, P-PDHE1A1 (S293), PDHE1A1, LDHA, G6PDH, and β-actin of protein extracts from control (CTRL) and NPRL2 KO primary astrocytes (*n* = 6 independent cultures per genotype). Quantification of protein abundance is shown as relative density (RD) normalized to β-actin. Each data point represents an independent biological culture. (**B**) Western blot analysis of P-S6K (T389), S6K, and β-actin from parental control and N3KO C8-D1A cell lines treated with EBSS for 1 h. Quantification of P-S6K (T389) normalized to S6K and β-actin. NPRL3, NPRL2, and β-actin western blots from selected lines are shown for validation. (**C**) QRT-PCR analysis of pyruvate dehydrogenase kinase isoforms (*Pdk1*, *Pdk2*, and *Pdk3*) in N3KO and control C8-D1A cells (*n* = 4 independent cultures per genotype). (**D**) QRT-PCR analysis of pyruvate dehydrogenase kinase isoforms (*Pdk1*, *Pdk2*, and *Pdk3*) from brain extracts of mice NPRL2 astroKO and CTRL at 5 months (*n* = 6 mice per genotype; biological replicates). (**E**) Quantification of NADPH/NADP+ ratio in CTRL and N3KO C8-D1A cells treated with or without dichloroacetate (DCA; 5 mM) or *n*-acetyl-l-cysteine (NAC; 5 mM) for 1 h. Each data point represents an independent biological culture (*n* = 3 cultures per genotype). (**F**–**H**) Representative metabolite profiling of N3KO and control C8-D1A cells, including: (**F**) phosphoenolpyruvate, (**G**) pyruvate, and (**H**) lactate (*n* = 3 independent cultures per genotype). All mass spectrometry was replicated at least two times in independent experiments. Data were shown as the mean ±  SEM. Welch’s *t*-test and one-way ANOVA with post hoc Tukey were used for comparisons.

**Figure 2:**
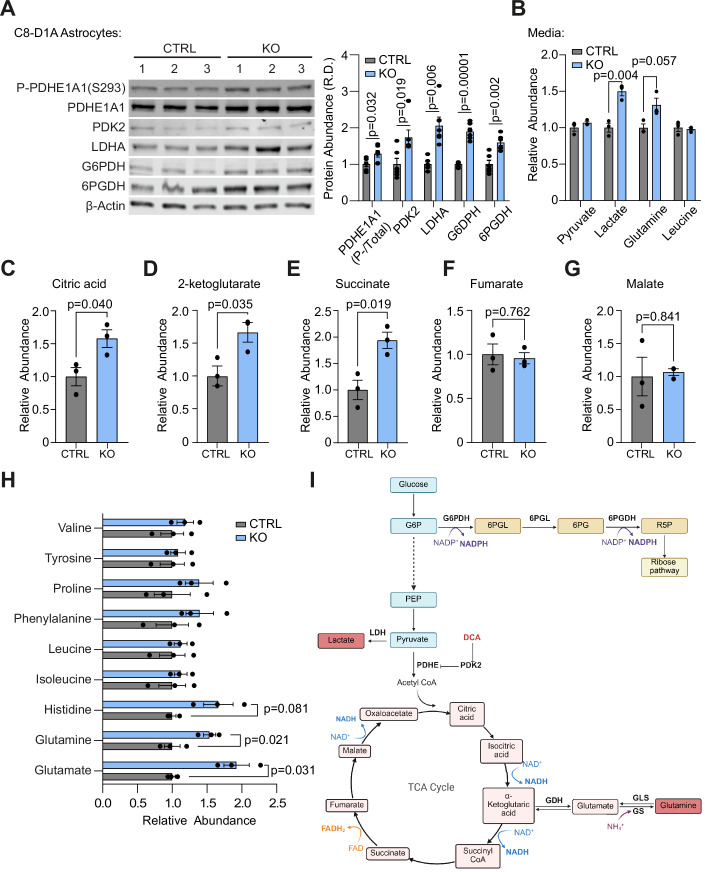
Metabolic rewiring in GATOR1-deficient astrocytes. (**A**) Representative western blot analysis of P-PDHE1A1 (S293), total PDHEA1, PDK2, LDHA, G6PDH, 6PGDH, and β-actin in control (CTRL) and N3KO C8-D1A cells. Each lane represents an independent biological culture (*n* = 3 per genotype shown). Quantification compiled from *n* = 6 independent biological cultures per genotype. Relative density (RD) of P-PDHE1A1 was normalized to total PDHE1A1 and β-actin; PDK2, LDHA, G6PDH, and 6PGDH normalized to β-actin. (**B**) Targeted-metabolite profiling of conditioned media from CTRL and N3KO cells (*n* = 3 independent cultures per genotype). (**C**) Quantification of intracellular citric acid cycle metabolites from CTRL and N3KO C8-D1A cell extracts, including (**D**) 2-ketoglutarate, (**E**) succinate, (**F**) fumarate, and (**G**) malate (*n* = 3 independent cultures per genotype). (**H**) Relative abundance of intracellular amino acids from metabolite extracts of CTRL and N3KO C8-D1A cells (*n* = 3 independent cultures per genotype). (**I**) Schematic representation of glycolysis, the pentose phosphate pathway (PPP), the TCA cycle, and glutamine biosynthesis pathways. All mass spectrometry experiments were performed in two independent biological experiments with consistent results. Data were shown as mean ± SEM. Welch’s *t*-test was used for statistical comparison. Source data are available online for this figure.

We previously showed that NPRL2 deletion alters mitochondrial carbohydrate metabolism by inducing a Warburg-like phenotype in skeletal muscle (Dutchak et al, 2018). To test whether GATOR1 loss similarly reprograms astrocytic metabolism, we isolated and cultured primary hippocampal astrocytes from NPRL2 astroKO and control mice. Western blot analysis of primary astrocyte protein extracts revealed a statistically significant increase in phosphorylated S6 and pyruvate dehydrogenase complex subunit E1alpha1 (PDHE1A1), the rate-limiting enzyme of pyruvate entry into the mitochondria (Fig. EV2A). Expression of lactate dehydrogenase (LDHA) and glucose-6-phosphate dehydrogenase (G6PDH), the rate-limiting enzyme of the pentose phosphate pathways (PPP), was also significantly elevated in the NPRL2 KO astrocytes compared to the control. To extend these observations using an astrocytic cell-line model, we generated immortalized NPRL3 KO (N3KO) C8-D1A cells (ATCC, CRL-2541) using our validated CRISPR-mediated NPRL3 KO strategy, as previously (Alliot and Pessac, 1984; Muller et al, 2024). Notably, loss of either NPRL2 or NPRL3 blocks GATOR1 complex function in cells with similar downstream impacts on mTORC1 signaling (Bar**-**Peled et al, 2013). Under strict starvation conditions using Earle’s buffered saline solution (EBSS), which lacks both amino acids and growth factors, C8-D1A N3KO cells failed to suppress S6K phosphorylation to parental control levels (Fig. EV2B). N3KO cells also showed increased inhibitory phosphorylation of PDHE1A1 at Ser293, together with higher pyruvate dehydrogenase kinase 2 (PDK2) and LDHA expression (Fig. 2A). Additionally, N3KO cells expressed significantly more G6PDH and 6-phosphogluconate (6PGDH), indicating enhanced pentose phosphate pathway (PPP) utilization, a major source of NADPH for anabolic metabolism and glutathione-dependent antioxidant defense. These observations are consistent with mTORC1-dependent regulation of carbohydrate metabolism and cellular redox state (Saxton and Sabatini, 2017). To further examine the regulation of pyruvate metabolism, we performed QRT-PCR analysis of pyruvate dehydrogenase kinase isoforms in both C8-D1A cells and brain extracts, and observed an increased expression of *Pdk2* mRNA in the knockouts (Fig. EV2C,D). To test whether GATOR1 deficiency could influence cellular redox homeostasis, we measured NADPH/NADP+ ratios in control and N3KO cells treated with or without dichloroacetate (DCA; a PDK inhibitor) or N-acetylcysteine (NAC; an antioxidant). Under basal conditions, N3KO cells showed a significantly reduced NADPH/NADP+ ratio compared to control, which was restored to control levels following either DCA or NAC treatment (Fig. EV2E). These findings support a metabolic rewiring phenotype in GATOR1-deficient astrocytes, consistent with a Warburg-like effect. Despite the upregulation of PPP enzymes, the reduced NADPH/NADP+ ratio suggests that endogenous antioxidant defenses that depend on NADPH, including glutathione, are redox-compromised to counteract any GATOR1-dependent redox stress, which may contribute to damage within the astrocytes or neighboring cells over time (Meister, 1988).

Within the brain, astrocytes support neurons by providing key metabolites to help maintain energy homeostasis and support synaptic functions. To determine if GATOR1 defects changed the efflux of metabolites from astrocytes, we used a targeted gas-chromatography/mass spectrometry (GC-MS) analysis of conditioned cell culture media. Our results showed significantly more extracellular lactate and elevated glutamine in the media from N3KO astrocytes cultures compared to controls (Fig. 2B). These data prompted us to investigate the intracellular metabolic status of the cells. Targeted GC-MS analysis of N3KO metabolite extracts showed approximately twofold higher intracellular lactate, with no change in phosphoenolpyruvate or pyruvate (Fig. EV2F–H). Intriguingly, TCA cycle intermediates from citrate to succinate were also elevated approximately twofold, while fumarate and malate were unchanged compared to controls (Fig. 2C–G). Most amino acid levels were not significantly different, however glutamine, glutamate were significantly increased in the N3KO cells (Fig. 2H). Collectively, these results show that defective GATOR1 function in astrocytes increases extracellular glutamine and lactate, which are known to support neuronal function (Fig. 2I). Importantly, the accumulation of succinate, without a corresponding increase in downstream metabolites like fumarate and malate, is consistent with impaired succinate dehydrogenase (SDH) activity that connects TCA cycle metabolism with electron transport chain (ETC) activity at Complex II (CII) (Tretter et al, 2016). Consistent with this, SDH dysfunction has been reported to reroute TCA cycle metabolism and enhanced glutamine synthesis under ETC-compromised conditions (Benit et al, 2022; Vicente-Gutierrez et al, 2019). These data support that astrocytic GATOR1 coordinates intracellular metabolite pools that supply of extracellular lactate and glutamine to support neuronal activity.

### Mitochondrial electron transport chain defects in GATOR1-defective astrocytes

**Figure 3:**
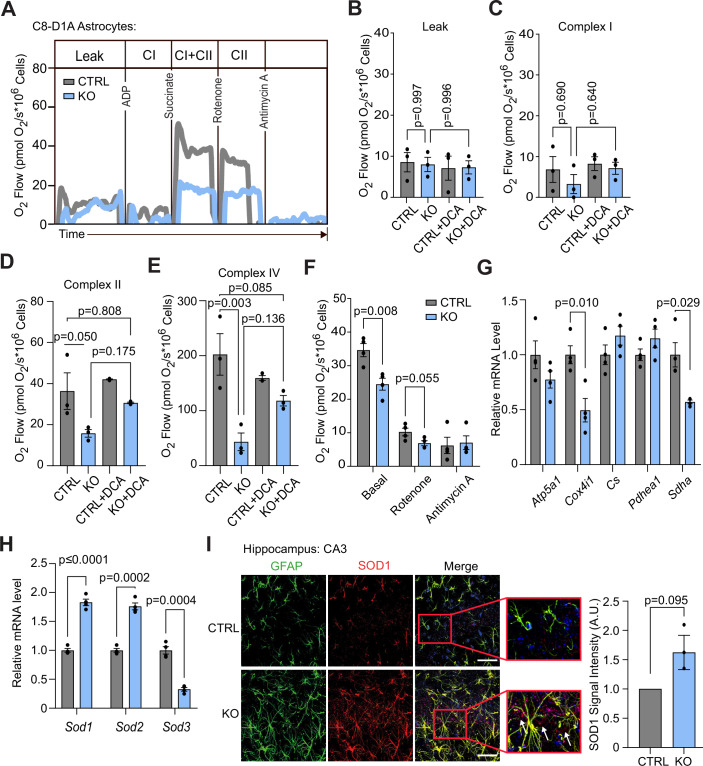
Electron transport chain impairment in GATOR1-deficient astrocytes. (**A**) Representative oxygen consumption tracings of permeabilized control (CTRL) and N3KO C8-D1A cells measured using an Oroboros O2k high-resolution respirometer. (**B**–**E**) Quantification of oxygen consumption corresponding to (**B**) leak respiration, (**C**) Complex I-linked respiration, (**D**) Complex II-linked respiration, and (**E**) Complex IV-linked respiration in CTRL and N3KO C8-D1A cells, treated with or without dichloroacetate (DCA; 5 mM; 16 h). Each data point represents an independent respirometer assay performed on independently prepared cultures (*n* = 3 independent cultures per condition, prepared on separate days, and analyzed in independent respirometry assays). (**F**) Basal oxygen consumption and response to rotenone and antimycin A in intact cells. Each data point represents an independent respirometer assay performed on independently prepared cultures (*n* = 4 independent cultures per condition). (**G**) QRT-PCR analysis of mitochondrial genes (*Atp5a1, Cox4i1*, *Cs, Pdhe1a1,* and *Sdha*) in CTRL and NPRL3 KO C8-D1A astrocytes (*n* = 4 independent cultures per genotype). (**H**) QRT-PCR analysis of *Sod1*, *Sod2*, and *Sod3* expression in control and N3KO C8-D1A cells (*n* = 4 independent cultures per genotype). (**I**) Representative confocal microscopy images of the hippocampal CA3 region at 5 months, along with a zoomed-in cross-sectional area stained for GFAP, SOD1, and DAPI with quantification of SOD1 signal intensity (*n* = 3 independent biological experiments; values represent KO normalized to matched WT within each experiment; two-tailed one-sample *t-*test vs 1 AU, arbitrary units). Scale bars, 40 µm. Data were shown as the mean ± SEM. Welch’s *t*-test and one-way ANOVA with post hoc Tukey were used for comparisons. Source data are available online for this figure.

The altered metabolic profile of TCA cycle intermediates in the N3KO cells prompted us to assess mitochondrial ETC activity using high-resolution respirometry (Oroboros O2k). To test if enhanced pyruvate oxidation could mitigate potential defects in ETC activity, N3KO and control cells were also treated with or without DCA for comparative purposes. In permeabilized cells, electron leak was not different between genotypes (Fig. 3A,B) and the addition of CI substrate, adenosine diphosphate (ADP), did not differentially affect oxygen consumption (Fig. 3A,C). In contrast, the subsequent addition of succinate, the CII substrate, increased oxygen consumption in control cells, but elicited a blunted response in N3KO cells, before or after the addition of rotenone (Fig. 3A,D). These CII-dependent respiration defects are consistent with our mass spectrometry measurements showing an accumulation of succinate, but not fumarate or malate, in the N3KO cells (Fig. 2E–G). Complex IV-dependent respiration was also significantly lower in N3KO cells compared to control (Fig. 3E). Notably, DCA pretreatment restored CII activity to control cell levels, and partially rescued CIV function (Fig. 3D,E). These results indicate that GATOR1 defects impair CII- and CIV-dependent mitochondrial respiration, which can be partially rescued by enhancing pyruvate flux. In intact cells, basal respiration driven by the combined activity of CI and CII was significantly lower in N3KO cells, and further decreased by rotenone, which isolates CII-dependent respiration (Fig. 3F). Since this functional effect was consistent with our metabolite analysis, we used QRT-PCR to measure the expression of key mitochondrial genes including: ATP synthase F1 subunit alpha (*ATP5a1*), cytochrome c oxidase subunit 4 isoform 1 (*Cox4i1*), citrate synthase (*Cs*), *Pdhe1a1*, and succinate dehydrogenase A (*Sdha*). Both *Cox4i1* and *Sdha* were significantly reduced in N3KO cells compared to control, consistent with impaired electron flow through both CII and the terminal step of the electron transport chain (Fig. 3G). These transcriptional changes further support a coordinated mitochondrial response to GATOR1 deficiency, which contributes to the deficit in respiration capacity and redox stress.

**Figure EV3:**
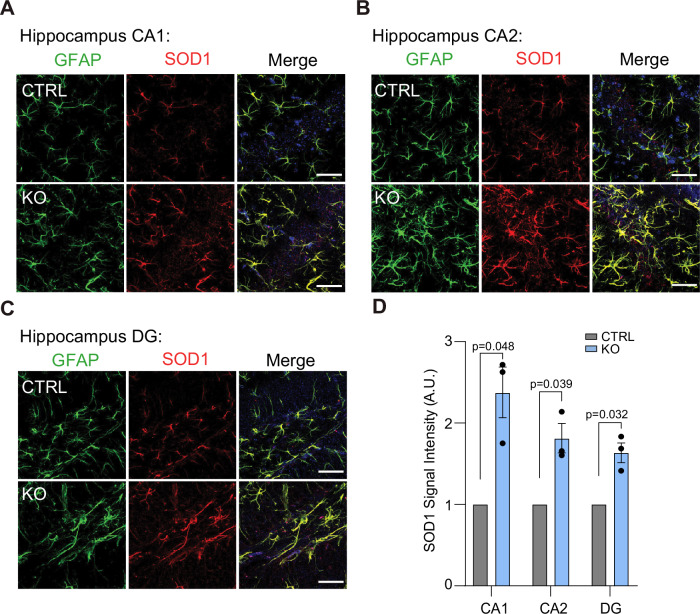
Astrocytic GATOR1-deficiency increases hippocampal SOD1 expression. (**A**–**C**) Representative confocal microscopy images of the hippocampal (**A**) CA1, (**B**) CA2, and (**C**) dentate gyrus (DG) regions stained for GFAP, SOD1, and DAPI. Scale bars = 40 µm. (**D**) Quantification of SOD1 signal intensity (arbitrary units, AU) in the indicated hippocampal regions. (*n* = 3 independent biological experiments; values represent KO normalized to matched WT within each experiment; two-tailed one-sample *t-*test vs 1 AU, arbitrary units). Data were shown as the mean ± SEM.

To test whether ETC defects trigger an adaptive antioxidant response, we measured the expression of superoxide dismutase (SOD), namely *Sod1*, *Sod2*, and *Sod3* mRNA by QRT-PCR in N3KO and control cells. The results showed a strong upregulation of *Sod1* and *Sod2* mRNA in N3KO cells relative to controls (Fig. 3H). IHC and confocal microscopy further showed an increase of SOD1 expression in GFAP-positive astrocytes and neighboring cells in the NPRL2 astroKO hippocampus (Figs. 3I and EV3A–D). Collectively, these findings indicate that GATOR1 deficiency leads to ETC defects and a stimulation of compensatory antioxidant programs, likely in response to sustained mitochondrial redox stress.

### GATOR1 defects enhance mitochondrial ROS production in an mTORC1-dependent manner

**Figure 4:**
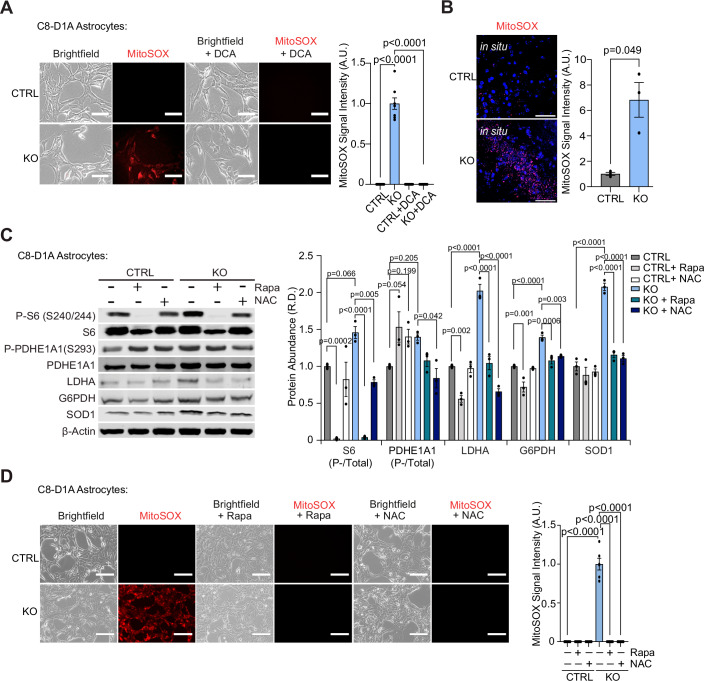
GATOR1-deficient astrocytes exhibit increased mitochondrial ROS and redox imbalance. (**A**) Representative epifluorescence images of control (CTRL) and N3KO C8-D1A cells stained with MitoSOX Red for 15 min to assess mitochondrial superoxide levels in the absence or presence of dichloroacetate (DCA; 5 mM, 2 h). Quantification of MitoSOX Red fluorescent signal intensity is shown (*n* = 8 independent cultures per condition; AU, arbitrary units). Scale bar, 75 µm. (**B**) Representative fluorescent images of hippocampal CA3 sections from control and NPRL2 astroKO mice stained with MitoSOX Red (30 min), with quantification of MitoSOX Red fluorescent signal intensity (*n* = 3 mice per genotype; biological replicates). Scale bar, 40 µm. (**C**) Western blot analysis of P-S6 (S240/244), total S6, P-PDHE1A1 (S293), total PDHE1A1, LDHA, G6PDH, SOD1, and β-actin in control and N3KO C8-D1A cells treated for 24 h with rapamycin (10 nM) or *N*-acetylcysteine (NAC; 5 mM). Quantification of protein abundance is shown as relative density (R.D.) normalized to β-actin. Each data point represents an independent biological experiment (*n* = 3 independent biological cultures). (**D**) Representative images of MitoSOX Red-stained C8-D1A cells following 24-h treatment with NAC (5 mM) or rapamycin (10 nM), with quantification of fluorescent intensity (*n* = 6 independent cultures per condition; AU, arbitrary units). Scale bar = 125 µm. Data were shown as mean  ± SEM. Welch’s *t*-test and one-way ANOVA with post hoc Tukey were used for statistical comparisons. Source data are available online for this figure.

**Figure EV4:**
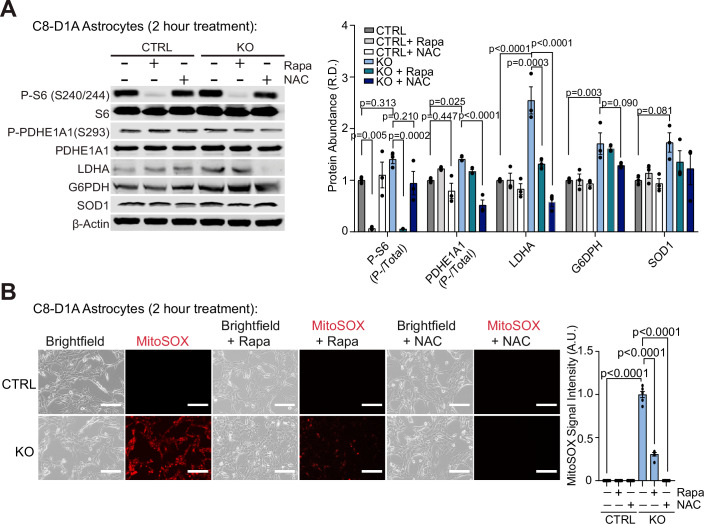
Rapamycin and antioxidant treatment ameliorate GATOR1-dependent ROS. (**A**) Representative western blot analysis of P-S6 (S240/244), S6, P-PDHE1A1 (S293), PDHE1A1, LDHA, G6PDH, SOD1, and β-actin from control (CTRL) and N3KO C8-D1A cells treated for 2 h with rapamycin (10 nM) or *n*-acetyl-l-cysteine (NAC; 5 mM) (*n* = 3 independent cultures per condition). Quantification of protein abundance is shown as relative density (R.D.), with phospho-protein levels standardized to their respective total protein levels and then normalized to β-actin. (**B**) Representative fluorescence images of CTRL and N3KO C8-D1A cells stained with MitoSOX Red for 15 min following pretreatment with rapamycin (10 nM) or NAC (5 mM) for 2 h, with quantification of fluorescent signal intensity (AU, arbitrary units). Each data point represents an independent biological culture (*n* = 6 cultures per genotype; biological replicates). Scale bar = 125 µm. Data were shown as mean ± SEM. One-way ANOVA with post hoc Tukey were used for comparison.

Complex II dysfunction can result in premature electron transfer to oxygen, generating superoxide, a primary form of reactive oxygen species (ROS) (Hadrava Vanova et al, 2020). To assess ROS production in our model, we stained control and N3KO C8-D1A cells with MitoSOX Red, a fluorogenic dye selective for mitochondrial superoxide. N3KO cells showed a marked increase in MitoSOX fluorescence, which was reduced to control levels following 16 h treatment with DCA (Fig. 4A), consistent with DCA enhancing the metabolic flux toward CII activity (Fig. 3D) (Pfleger et al, 2015). Acute hippocampal brain slices from NPRL2 astroKO also displayed approximately sixfold higher MitoSOX fluorescence relative to controls (Fig. 4B). The ability of DCA to suppress ROS supports a direct link between pyruvate flux to ETC efficiency and redox regulation in GATOR1-deficient astrocytes. To test whether antioxidant or mTORC1-targeted interventions act through overlapping mechanisms, we treated N3KO and control cells with *N*-acetyl-l-cysteine (NAC) or rapamycin for 2 or 24 h (Fig. EV4A,C). Rapamycin treatment suppressed P-S6 in both genotypes, confirming mTORC1 inhibition. NAC selectively reduced phosphorylation of PDHE1A1 (Ser293) in N3KO cells, while unexpectedly increasing it in controls after 24 h; both treatments also normalized LDHA in N3KO cells, but only rapamycin reduced LDHA in controls. G6PDH expression, reflecting oxidative PPP flux, was downregulated by rapamycin in both genotypes at 24 h, but only reduced by NAC in N3KO cells at both time points. Of note, SOD1 was selectively upregulated in untreated N3KO cells, but was significantly reduced 24 h after rapamycin or NAC treatment. We also performed parallel experiments to determine how these treatments affected mitochondrial ROS production. Using MitoSOX assays, we found that short-term (2 h) rapamycin treatment produced a modest, but statistically significant reduction in ROS in the N3KO cells, whereas NAC fully restored ROS to control levels over the same period (Fig. EV4B,D). At 24 h, rapamycin or NAC treatment was sufficient to completely suppress the elevated mitochondrial ROS in the N3KO cells to control levels. These findings support that antioxidants can rapidly normalize GATOR1-dependent ROS in cells, while sustained mTORC1 inhibition can indirectly restore redox balance over a longer time. Taken together, these results suggest that oxidative stress, and not only mTORC1 signaling, contributes to the metabolic rewiring in GATOR1-deficient astrocytes. In this context, the elevated ROS and metabolic alterations, previously linked to CII dysfunction and redox imbalance (Vicente-Gutierrez et al, 2019), reinforce a maladaptive, pro-excitatory metabolic state that can support neural circuit hyperexcitability.

### GATOR1 defects enhance local glutamine supply for neurotransmission

**Figure EV5:**
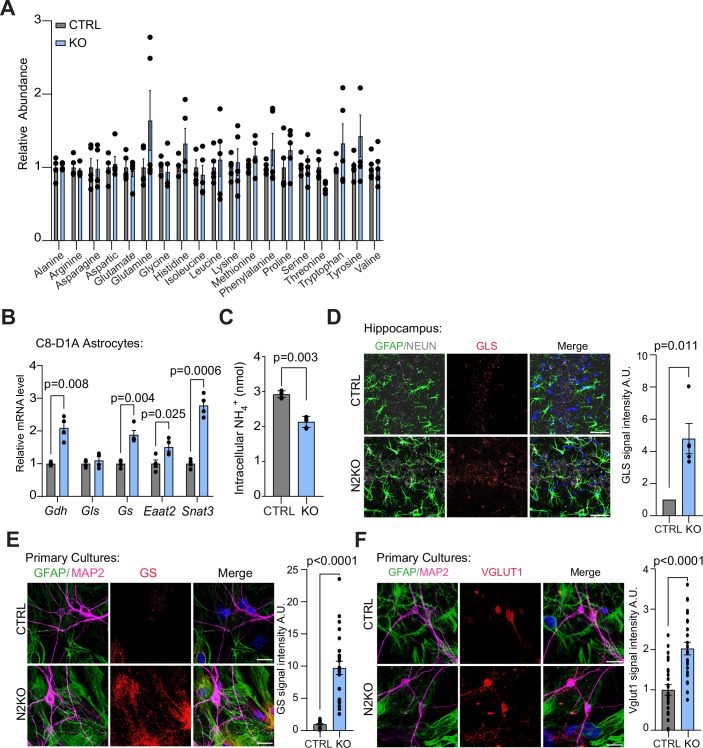
GATOR1 defective astrocytes stimulate neuronal gene expression. (**A**) Metabolomic analysis of relative amino acid abundance in brain extracts from NPRL2 astroKO and control (CTRL) mice at 2 months of age (*n* = 5 mice per genotype; biological replicates). (**B**) QRT-PCR analysis of metabolic enzymes (*Gdh*, *Gls,* and *Gs*) and astrocytic amino acid transporters (*Eaat2* and *Snat3*) from CTRL and N3KO C8-D1A cells (*n* = 4 cultures, independent cultures per genotype). (**C**) Ammonia (NH4+) concentration measured in CTRL and N3KO C8-D1A cells (*n* = 3 independent cultures per genotype). (**D**) Representative confocal microscopy images from 5-month-old CTRL and NPRL2 astrocKO mice stained for GFAP, NEUN, glutaminase (GLS), and DAPI. Quantification of GLS signaling intensity relative to CTRL is shown (*n* = 5 independent biological experiments; values represent KO normalized to matched WT within each experiment; two-tailed one-sample *t-*test vs 1 AU, arbitrary units). Scale bar = 40 μm. (**E**) Representative confocal microscopy images of co-cultures of control neurons with CTRL or NPRL2 KO primary astrocytes, stained for GFAP, MAP2, GS, and DAPI, with quantification of GS signal intensity in astrocytes (*n* = 5 individual cultures per genotype; 5 fields quantified per culture). Scale bar = 20 μm. (**F**) Representative confocal microscopy images of control neurons with CTRL or NPRL2 KO primary astrocytes, stained for GFAP, MAP2, VGLUT1, and DAPI. Quantification of vesicular glutamate transporter (VGLUT1) signal intensity in neurons (*n* = 4 individual cultures per genotype; 5 fields quantified per culture). Scale bar = 20 μm. Data were shown as the mean ± SEM. Welch’s *t*-test was used for statistical comparisons.

**Figure 5:**
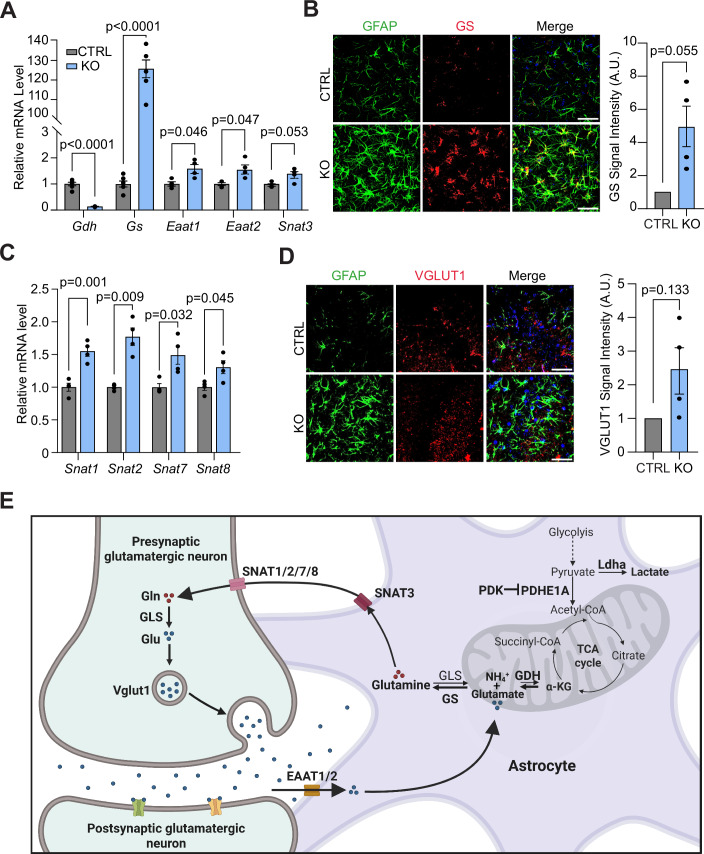
GATOR1 deficiency enhances astrocyte-neuron glutamate-glutamine coupling. (**A**) QRT-PCR analysis of metabolic enzymes (*Gdh* and* Gs*) and astrocytic amino acid transporters (*Eaat1*, *Eaat2*, and *Snat3*) in brain extracts from NPRL2 astroKO and control (CTRL) mice at 5 months (*n* = 4–6 mice per genotype, depending on target gene; biological replicates). (**B**) Representative confocal microscopy images of hippocampal CA3 sections at 5 months, stained for GFAP, glutamine synthetase (GS), and DAPI with quantification of GS signal intensity (*n* = 4 independent biological replicates; values represent KO normalized to matched WT within each experiment; two-tailed one-sample *t-*test vs 1 AU, arbitrary units). (**C**) QRT-PCR analysis of neuronal glutamine transporters (*Snat1*, *Snat2*, *Snat7*, and *Snat8*) in whole-brain extracts from NPRL2 astroKO and CTRL mice at 5 months (*n* = 4 mice per genotype; biological replicates). (**D**) Representative confocal microscopy images and quantification of hippocampal CA3 sections stained for GFAP, VGLUT1 and DAPI (*n* = 4 independent biological replicates; values represent KO normalized to matched WT within each experiment; two-tailed one-sample *t-*test vs 1 AU, arbitrary units). Scale bar = 40 µm. (**E**) Schematic model illustrating how GATOR1 deficiency alters the glutamate-glutamine cycle between astrocytes and neurons. Data were shown as the mean ± SEM. Welch’s *t*-test was used for statistical comparisons. Source data are available online for this figure.

To test whether astrocytic GATOR1 dysfunction alters whole-brain metabolism, we performed liquid chromatography-mass spectrometry (LC-MS/MS) metabolomics on pre-symptomatic brain extracts from 2-month old NPRL2 astroKO and littermate control mice, aiming to capture early metabolic changes prior to overt astrocytic reactivity and seizure onset (Absolute*ID* p180 kit; Biocrates Life Sciences). Most amino acids were similar between genotypes, yet NPRL2 astroKO brains showed a trend toward increased glutamine content (Fig. EV5A), consistent with elevated glutamine in N3KO conditioned media and cells (Fig. 2B,H). To determine if these in vivo metabolic changes were transcriptionally mediated, we used QRT-PCR to measure the expression of key genes involved in glutamine metabolism. QRT-PCR analysis revealed significant upregulation of glutamine synthetase (GS) in the brain (Fig. 5A), as well as GATOR1-deficient cells (Fig. EV5B); whereas glutamate dehydrogenase (*Gdh*) was reduced only in the tissue, likely reflecting non-astrocytic cell populations. IHC and confocal microscopy confirmed approximately fivefold higher GS abundance in GFAP-positive astrocytes of the NPRL2 astroKO hippocampus compared to controls (Fig. 5B). This upregulation of GS suggested that GATOR1-deficient astrocytes could increase glutamine production through enhanced glutamate-ammonium coupling. Consistent with this reaction, intracellular ammonium, the nitrogen donor for GS-mediated glutamine synthesis, was found to be reduced in N3KO cells (Fig. EV5C). Collectively, these data suggest that GATOR1 deficiency promotes GS-mediated glutamate to glutamine conversion in astrocytes to increase local glutamine supply.

To test whether altered glutamine homeostasis influenced glutamate handling, we examined the expression of their transporters in GATOR1-deficient cells and tissues using QRT-PCR. Astrocytic glutamate uptake transporters *Eaat1* and *Eaat2*, along with the glutamine efflux transporter *Snat3*, were modestly but significantly increased in GATOR1-deficient brain extracts (Figs. 5A and EV5B). The expression profile of these transporters supports an astrocyte-autonomous program for glutamate uptake and glutamine export under conditions of chronic mTORC1 activation. Moreover, in the brain, neuronal glutamine transporters *Snat1*, *Snat2*, *Snat7* and Snat8 were also upregulated, supporting an increased capacity for glutamine intake into the neurons (Fig. 5C). Consistent with these differences, IHC showed an increased abundance of glutaminase (GLS) expression in cells adjacent to GFAP-positive astrocytes in NPRL2 astroKO hippocampus, with NeuN co-staining identifying them as neurons (Fig. EV5D). These results suggest that neurons respond to increased astrocytic glutamine availability by upregulating glutamine uptake and catabolism into glutamate. We also observed a trend for increased abundance of vesicular glutamate transporter 1 (VGLUT1) immunostaining in the NPRL2 astroKO tissue, consistent with glutamate packing into synaptic vesicles to support excitatory neuronal communication (Fig. 5D). We followed these in vivo studies with co-culture experiments using primary neurons from control mice, grown with either primary NPRL2 KO astrocytes or control astrocytes. In culture, NPRL2 KO primary astrocytes also expressed significantly more GS protein (Fig. EV5E), with adjacent neurons expressing approximately twofold higher VGLUT1 compared to neurons with control astrocytes (Fig. EV5E,F), mirroring the in vivo observations. These data suggest that GATOR1-deficient astrocytes induce coordinated remodeling of glutamine synthesis and glutamate packaging, consistent with a greater capacity for loading glutamate into synaptic vesicles (Fig. 5E). We propose this remodeling may amplify glutamate-glutamine cycling and promote a pro-excitatory network state, contributing to seizure susceptibility in GATOR1-related disorders.

Collectively, these findings demonstrate that GATOR1 is a critical regulator of astrocytic metabolism, redox balance, and neuron-astrocyte metabolic coupling. Loss of GATOR1 impairs mitochondrial Complex II function and antioxidant capacity, triggers glutamine biosynthesis and enhances glutamate-glutamine cycling pathways. This metabolic rewiring promotes increased neuronal glutamate packaging and synaptic loading, establishing a pro-excitatory state that likely contributes to seizure susceptibility. These data position astrocytic GATOR1 as a novel and central link between nutrient sensing and circuit-level excitability in mTORC1-driven neurological disorders and offers an explanation to clarify why standard anti-seizure therapies, such as sodium channel inhibitors, are insufficient in many patients with GATOR1 mutations, as seizures in this context arise not only from intrinsic neuronal hyperexcitability but from altered astrocyte metabolism.

## Methods

**Table:** Reagent and tools table

Reagent/resource	Reference or source	Identifier or catalog number
**Experimental models**		
C57/B6 (M. musculus)	Jackson Laboratory	#00664
*Gfap-ires-*Cre	Jackson Laboratory	#02498
NPRL2^fl/fl^	Dutchak, et al 2015	
C8-D1A cells	ATCC	CRL-2541
**Oligonucleotide primers**		
Atp5a1 F1		5’GTCTCTCCGAGAAGCTGCAA3’
Atp5a1 R1		5’CATTTTTGGAGACCAGTCCCG3’
Cox4i1 F1		5’GCCTTGGACGGCGGAATG3’
Cox4i1 R1		5’CGAAGGCACACCGAAGTAGA3’
Cs F1		5’GCAGCAACATGGGAAGACAGT3’
Cs R1		5’CCTCTCATGCCACCGTACAT3’
Eaat1 F1		5’CAGTCTCGTCACAGGAATGGC3’
Eaat1 R1		5’ATAGACTACAGCGCGCATCC3’
Eaat2 F1		5’ATCAACAGAGGGTGCCAACA3’
Eaat2 R1		5’CTTTGGCTCATCGGAGCTGA3’
Gdh F1		5’GAGGTCATCGAAGGCTACCG3’
Gdh R1		5’TGCTGTAACGGATACCTCCC3’
Gls F1		5’CCGGGCCCCAAGGACA3’
Gls R1		5’ACCCTGTTTTATTTTATTGTCGCC3’
Gs F1		5’CACCCCTGGTTTGGAATGGA3’
Gs R1		5’CAGTAATACGGGCCTTGGGG3’
Pdhe1a1 F1		5’GACTCAGGGTAGATGGAATGGAT3’
Pdhe1a1 R1		5’GGCAGCCGCAAACTTTGT3’
Pdk1 F1		5’TGCAAAGTTGGTATATCCAAAGCC3’
Pdk1 R1		5’TGTGCCGGTTTCTGATCCTT3’
Pdk2 F1		5’GCGCTGTTGAAGAATGCGT3’
Pdk2 R1		5’GCATTGCTGGATCCGAAGTCT3’
Pdk3 F1		5’TCCTGGACTTCGGAAGGGAT3’
Pdk3 R1		5’GTTAGCCAGTCGCACAGGAA3’
Sdha F1		5’TATTGCTACTGGGGGCTACG3’
Sdha R1		5’CCCTAGTGACCATGGCTGTG3’
Snat1 F1		5’TCCCTAGCTAGTCCTTGACAC3’
Snat1 R1		5’GCCTGAGACCCCTCGAGAA3’
Snat2 F1		5’GGAGAGCACCGAGGCAG3’
Snat2 R1		5’CTTCATGCTGCTCGGGAGG3’
Snat3 F1		5’CACCACGAGGCCAGATACC3’
Snat3 R1		5’CACCATCTCTGTCTGTCGGG3’
Snat7 F1		5’ACTTGAGGCCAAAAGGGGAG3’
Snat7 R1		5’GGGAACTCTTGTCAACCTGCT3’
Snat8 F1		5’AGCTGCTGCCCATGATCC3’
Snat8 R1		5’GCATGCAAAACCAGCGCA3’
Sod1 F1		5’GGGAAGCATGGCGATGAAAG3’
Sod1 R1		5’GGTTCACCGCTTGCCTTCTG3’
Sod2 F1		5’TGCAAGGAACAACAGGCCTTA3’
Sod2 R1		5’TACTGAAGGTAGTAAGCGTGCTC3’
Sod3 F1		5’GCAACTCAGAGGCTCTTCCT3’
Sod3 R1		5’TGAGGTTCTCTGCACCTGTC3’
Genotyping Nprl2 F1		5′CTCAGGTTCTACGCAGTGACTTC3′
Genotyping Nprl2 R1		5′CATGGCGCTGTCTGGATCC3′
Genotyping Nprl2 knockout F1		5′CAGGCTTCATACTTCTACCCTC3′
Genotyping Gfap F1		5′TCCATAAAGGCCCTGACATC3′
Genotyping Gfap R1		5′TGCGAACCTCATCACTCGT3′
**Antibodies**		
6PGDH	Proteintech	147181-AP
β-actin	CST	3700S
CRE	CST	15036
G6PDH	Proteintech	254131-AP
GFAP	Millipore Sigma	AB5541
GLS	Abcam	AB156876
GS	CST	80636
LDHA	CST	3582S
MAP2	Millipore Sigma	MAB3418 S
NEUN	Millipore Sigma	MAB377
NPRL2	Santa Cruz Biotechnology	sc-376986
NPRL3	Thermo Fisher Scientific	PA5-78244
PDHE1A1 (S293)	CST	37115S
PDHE1A1	Abcam	AB168379
PDK2	Santa Cruz Biotechnology	SC-517284
P-S6 (S240/244)	CST	2215S
P-S6K (T389)	CST	9205S
S6	CST	2217S
S6K	CST	9202S
SOD1	Proteintech	10269-1-AP
VGLUT1	Millipore Sigma	AB5905
Secondary: Alexa Fluor 488 Goat anti-chicken	Thermo Fisher Scientific	A32931
Secondary: Alexa Fluor 546 Goat anti-mice	Thermo Fisher Scientific	A-11030
Secondary: Alexa Fluor 647 Goat anti-rabbit	Thermo Fisher Scientific	A-21245
Secondary: Alexa Fluor 647 Goat anti-guinea pig	Thermo Fisher Scientific	A-21450
Secondary: IRDye 680RD Goat anti-mice IgG	LICORbio	926-68070
Secondary: IRDye 800CW Goat anti-rabbit IgG	LICORbio	926-32211

### Generation of NPRL2 astroKO mice

Astrocyte-specific GATOR1-defective mice were generated by crossing *Nprl2*^*loxP/+*^ mice, with *Gfap-ires-*Cre female mice to generate heterozygous knockout animals. Subsequent crosses exclusively used female carriers of the GFAP-*ires-*Cre allele to avoid any male germline-dependent *Cre* expression (Jackson Laboratory, #02498), resulting in control (*Nprl2*^*loxP/+*^ and *Nprl2*^*loxP/loxP*^) and NPRL2 astroKO mice (*Nprl2*^*loxP/loxP*^; *Gfap-ires-*Cre) were used for experimental investigation. Genotypes of all animals were determined by using PCR prior to experimental implementation in a non-blinded manner, with primers found in the Reagent/Resource table. All animal experiments were approved by the Université Laval Committee on Ethics and Animal Research (Protocol #23-1382).

### Generation of NPRL3 KO C8-D1A astrocytes

The mouse astrocytic cell line C8-D1A was obtained from ATCC (CRL-2541) and maintained as recommended. This line has been characterized as an astrocyte-derived model, and has been widely used in studies of astrocytic biology. This cell line was used to generate NPRL3 KO cells using a pre-designed NPRL3 targeted CRISPR/Cas9 vector, as previously (Muller et al, 2024). Stable cells were selected by treating cells with puromycin (2 μg/mL) for 48 h, followed by colony isolation and expansion. Individual clones were screened for NPRL3 knockout and their response to starvation conditions.

### Cell culture and treatments

Cells were cultured in DMEM (Gibco, 11965) supplemented with 10% fetal bovine serum (FBS) and maintained at 37 °C in 5% CO_2_. For amino acid starvation, cells were washed once with phosphate-buffered saline (PBS) and incubated in Earle’s balanced salt solution (EBSS) medium for 1 h at 37 °C in 5% CO_2_. As indicated in the corresponding figure legends, cells were treated with either 10 nM rapamycin (Cayman Chemical Company) or 5 mM *N*-acetyl-L-cysteine (1PlusChem) for either 2 or 24 h. For reactive oxygen species assays, cells were incubated with 5 µM MitoSOX Red (Thermo Fisher) 15 min prior to epifluorescence imaging.

### Primary glia cultures

Glial cells were prepared from P0–P1 mouse hippocampus. Briefly, dissected hippocampi were enzymatically dissociated using papain (400 U, Sigma) in MEM containing L-cysteine, followed by mechanical trituration through decreasing gauge needles. Cells were plated in DMEM supplemented with 10% FBS and maintained at 37  °C in 5% CO₂. Medium was changed the following day and every 2–3 days thereafter. Glial cultures were split after 7 days in vitro (DIV) and used between DIV14–21 for co-culture experiments.

### Primary co-cultures

Primary cortical neurons from P0–P1 control mice were plated on PDL-coated coverslips using previously described methods (9). Dissociated neurons were cultured in Neurobasal medium supplemented with B27 (Thermo Fisher), L-glutamine (Fisher Scientific), and penicillin/streptomycin (Thermo Fisher). On day in vitro (DIV) 2, primary hippocampal glia from NPRL2 astroKO or control mice were added to the neurons at a final ratio of 1:4 plated cells. On DIV3, cultures were treated with 5 µM AraC (Sigma). On DIV21, cells were fixed with 4% paraformaldehyde (PFA) and used for immunohistochemistry.

### Video recordings

Mice were video-recorded individually (one per cage) over a 48-h period at 5 months of age using a Sony FDR-AX53 camcorder. No seizures were observed in control animals. In NPRL2 astroKO mice, seizure number and duration were quantified.

### Immunohistochemistry

Mice were anesthetized with ketamine-xylazine (100 and 10 mg/kg) and perfused with PBS prior to fixation with 4% paraformaldehyde (PFA). The brain was post-fixed in 4% PFA for 24 h at 4 °C, and transferred to a 30% sucrose solution for an additional 48 h at 4 °C. Tissues were sectioned at 40 µm using a microtome (Microm HM430, Thermo Fisher). Tissue sections were washed three times with PBS for 10 min, followed by a 1 h incubation in blocking solution containing 3% BSA, 5% normal goat serum (NGS), 0.3% Triton X-100, and 0.02% sodium azide in PBS. Sections were incubated overnight at 4 °C in primary antibodies diluted in blocking solution. The primary antibodies used for IHC are shown in the Reagent/Resource table. The next day, brain sections were washed three times with PBS for 10 min, followed by a 2 h incubation with appropriate Alexa Fluor-conjugated secondary antibodies diluted in PBS. Secondary antibodies used are shown in the Reagent/Resource table. Sections were washed three times for 10 min with PBS and mounted with VECTASHIELD® mounting media (Vector Laboratories, VECTH1200). Images were taken using a Zeiss LSM710 confocal microscope and analysed in Fiji ImageJ using the signal intensity measuring tool. GFAP-positive astrocytes were segmented using Otsu thresholding on the green channel with identical parameters across all images. Astrocyte reactivity was assessed by quantifying GFAP-positive area and integrated density per image. Protein expression within astrocytes was measured within GFAP-positive regions, while neuronal-associated signal was quantified in regions excluding GFAP-positive areas where indicated. For immunohistochemistry quantification, values from control samples were normalized within each experimental group and target, thereby setting the control average to 1. This normalization enabled direct comparison of KO samples to their corresponding control while minimizing technical variability between experimental replicates.

### Quantitative (q)RT-PCR analysis

Total RNA from tissues or cells was extracted using TRIzol (Invitrogen), following the manufacturer's protocol. QRT-PCR primers were selected to span exon-exon junctions and are listed in the Reagent/Resources table. RNA extracts were treated with DNase (Roche) and the High-Cap cDNA Reverse Transcription kit (Applied Biosystems) was used for cDNA synthesis. Each qRT-PCR condition contained 25 ng cDNA, 150 nM each primer pair, and 5 μl SYBR GreenER (Invitrogen) and were analyzed in triplicate using the QuantStudio 5 Real-Time PCR system (Applied Biosystems). Relative mRNA abundance was calculated using the comparative threshold cycle method using U36B4 as the internal control.

### Western blot analysis

Brain tissue and cells were harvested in lysis buffer containing 150 mM sodium chloride, 50 mM sodium fluoride, 100 μM sodium orthovanadate, 50 mM sodium pyrophosphate tetrabasic, 10 mM β-glycerophosphate, 5 mM EDTA, 5 mM EGTA, and 0.5% Triton X-100 in 10 mM HEPES (pH 7.4), supplemented with complete anti-protease cocktail (Roche). Cleared lysates were boiled in 1x Laemmli sample buffer and separated on SDS-PAGE for western blot analysis. Membranes were blocked in 5% BSA and probed overnight with primary antibodies at 4 °C. Primary antibodies used are listed in the Reagent/resource table. Membranes were washed in TBST and incubated with LICOR IRDye secondary antibodies, listed in the Reagent/Resource table, for 1 h at room temperature before being washed in TBST and imaged using the LI-COR Odyssey imaging system. Fluorescent scans (LI-COR) were acquired below saturation; phospho-signals were quantified as p-target/total-target and normalized to β-actin internal control. Signal intensity was analyzed by densitometry using Image Studio Lite software version 5.2.

### NADPH/NADP kit assay

NADPH/NADP+ ratios were measured using the NADP+/NADPH-Glo Assay kit (Promega, G9081) using the manufacturer's protocol. Where indicated in the figure legends, cells were treated with either DCA 5 mM or NAC 5 mM for 1 h (1P0035SO, 1PlusChem) prior to analysis.

### Ammonia (NH4^+^) kit assay analysis

Intracellular ammonia was measured using the ammonia Assay Kit (ab83360, Abcam, Cambridge, MA) according to the manufacturer’s instructions from control and NPRL3 KO cells.

### GC-MS analysis of metabolites

Gas chromatography/mass spectrometry was performed at the Metabolomics Innovation Resource, McGill University. Briefly, for conditioned media analysis, 20 µL aliquots were collected in 180 µL of 90% methanol and dried using a speed vacuum (SpeedVac SPD1030, Thermo Scientific). For intracellular metabolite analysis, C8-D1A cells were first rinsed with ice-cold saline, harvested in 80% methanol, and sonicated three times for 10 s with 30 s cooling intervals on ice. Metabolite extracts were centrifuged for 15 min at 4 °C, and supernatants were collected. Solvent was removed using a speed vacuum, and samples were stored at −80 °C until being shipped on dry ice for analysis at the Metabolomics Innovation Resource at McGill University. The GS-MS and analysis were performed as previously (Nandi et al, 2024), and quantified metabolite values were normalized to protein content per sample.

### Brain metabolite analysis

The cortex of NPRL2 astroKO and littermate controls were harvested from living animals at 2 months of age, showing no physical signs of stress, by cervical dislocation, rapid dissection, and snap freezing of the tissue in liquid nitrogen. Samples were stored at −80 °C, until they were processed at the Analytical Facility for Bioactive Molecules, The Hospital for Sick Children (Toronto, Canada). One hemisphere was subjected to metabolite extraction using the Absolute*IDQ* p180 kit (Biocrates Life Sciences), using the manufacturer’s protocol and samples were analyzed on a SCIEX 5500 QTRAP, as previously (Hui et al, 2022).

### Acute brain slices

Age-matched control and NPRL2 astroKO mice were decapitated, and the brain was rapidly harvested, excluding the cerebellum. The cerebrum was glued cut-face down to a platform and placed into an ice-cold, oxygenated NMDG-based ACSF slicing solution containing: 93 mM N-methyl-D-glucamine (NMDG), 0.25 mM KCl, 1 mM MgCl₂·6H₂O, 0.05 mM CaCl₂·2H₂O, 0.125 mM NaH₂PO₄·H₂O, 28 mM NaHCO₃, 2 mM HEPES, 8 mM glucose, 5 mM sodium ascorbate (NaC₆H₇O₆), 3 mM sodium pyruvate (C₃H₃NaO₃), 2 mM thiourea, and 2 mM kynurenic acid. The solution was adjusted to pH 7.3 with HCl and maintained at 300–310 mOsm. Brains were sectioned at 100 μm on a vibratome (vibration speed 0.12 mm/s) while continuously oxygenated (95% O₂/5% CO₂). After sectioning, slices were transferred to a recovery/incubation ACSF containing: 125 mM NaCl, 2.5 mM KCl, 1 mM MgCl₂·6H₂O, 2.5 mM CaCl₂·2H₂O, 1 mM NaH₂PO₄·H₂O, 26 mM NaHCO₃, 20 mM sucrose, 2.5 mM glucose, and 1 mM sodium pyruvate, adjusted to pH 7.4 (300–310 mOsm). Slices were incubated for 30 min in oxygenated solution before treatment with 1 µM MitoSOX Red or DMSO for 30 min, then fixed in 4% paraformaldehyde (PFA) for 10 min and imaged.

### Oroboros O2K measurements

As indicated by the manufacturer’s instructions, the Oroboros O2K instrument was calibrated with DMEM media with 10% FBS (media used for C8-D1A cells). Where indicated, C8-D1A cells were treated with 5 mM of dichloroacetate (DCA, 1PlusChem) for 16 h, and cell counts were normalized prior to oxygen consumption measurements.

#### Basal respiration

Cells were put into the chambers without any detergent, and the oxygen consumption was measured corresponding to the basal, then 0.5 µM of rotenone was added, and finally, antimycin A 2.5 µM has been added.

#### ETC complex measurements

Briefly, the cells were transferred to the Oroboros chambers and 100 ug/mL digitonin was added to permeabilize the cells. For leak measurements, 5 mM pyruvate, 2 mM malate, and 10 mM glutamate were added to the chambers. Adenosine diphosphate (2.5 mM) was added to stimulate Complex I (CI) activity. About 10 mM succinate was added to stimulate Complex I and Complex II at the same time, followed by the addition of 0.5 µM rotenone to quench Complex 1 activity specifically. Finally, non-mitochondrial residual oxygen consumption (ROX) was measured by adding 2.5 μM antimycin A. All respiratory states were corrected for measures of ROX. Complex IV activity was then measured by simultaneously adding 2 mM ascorbate and 500 μM *N*,*N*,*N*′,*N*′-tetramethyl-1,4-benzenediamine dihydrochloride. Measurements were corrected by subtracting the oxygen consumption ratio (OCR) measurement acquired after the addition of 100 mM sodium azide.

### Statistical analysis

Statistical analyses were performed using GraphPad Prism 10.4.1 (GraphPad). The statistical unit was defined as an independent biological replicate (individual mouse or independently prepared primary cell culture). For IHC comparison, each replicate experiment in which immunostaining was performed on tissues collected from distinct mouse harvests and processed at different times was considered an independent biological experiment; values were normalized to matched WT within each experiment, and a two-tailed one-sample t-test was used for comparison. For IHC values, cell-type restricted quantification (e.g., astrocytic or non-astrocytic compartments) was performed using channel-based masking, and identical thresholding parameters which were applied across all samples within each experiment. For two-group comparisons, Welch’s *t*-tests were applied. For experiments involving more than two groups (e.g., genotype vs treatment comparisons such as rapamycin, NAC, or DCA rescue), one-way ANOVA with Tukey’s post-hoc test was used. Western blot quantification was performed on phospho/total protein ratios, or target protein levels, normalized to β-actin, prior to statistical comparison. For qRT-PCR analyses, statistical testing was conducted on fold-change values calculated using the ΔΔCt method. Data were presented as mean ± SEM unless otherwise indicated. Exact *p* values and biological replicate numbers (*n*) are provided in the figure legends, and the numerical datasets are provided in Dataset EV1.

## Supplementary information

Peer Review File

Dataset EV1

Movie EV1

Source data Fig. 1

Source data Fig. 2

Source data Fig. 3

Source data Fig. 4

Source data Fig. 5

Microscopy Source Data - for all figures

Expanded View Figures

## Data Availability

This study includes no data deposited in external repositories. The source data of this paper are collected in the following database record: biostudies:S-SCDT-10_1038-S44319-026-00846-w.
