# Supplementary material for: GATOR1 signaling defects promote astrocytic metabolic rewiring and excitatory neurotransmitter cycling
Source: EMBO Rep. 2026 Jul 3;27(15):4252–71. doi: 10.1038/s44319-026-00846-w (PMC13458053; doi:10.1038/s44319-026-00846-w)
Supplement: Supplementary file 1 [file 44319_2026_846_MOESM10_ESM.pdf]

Expanded View Figures

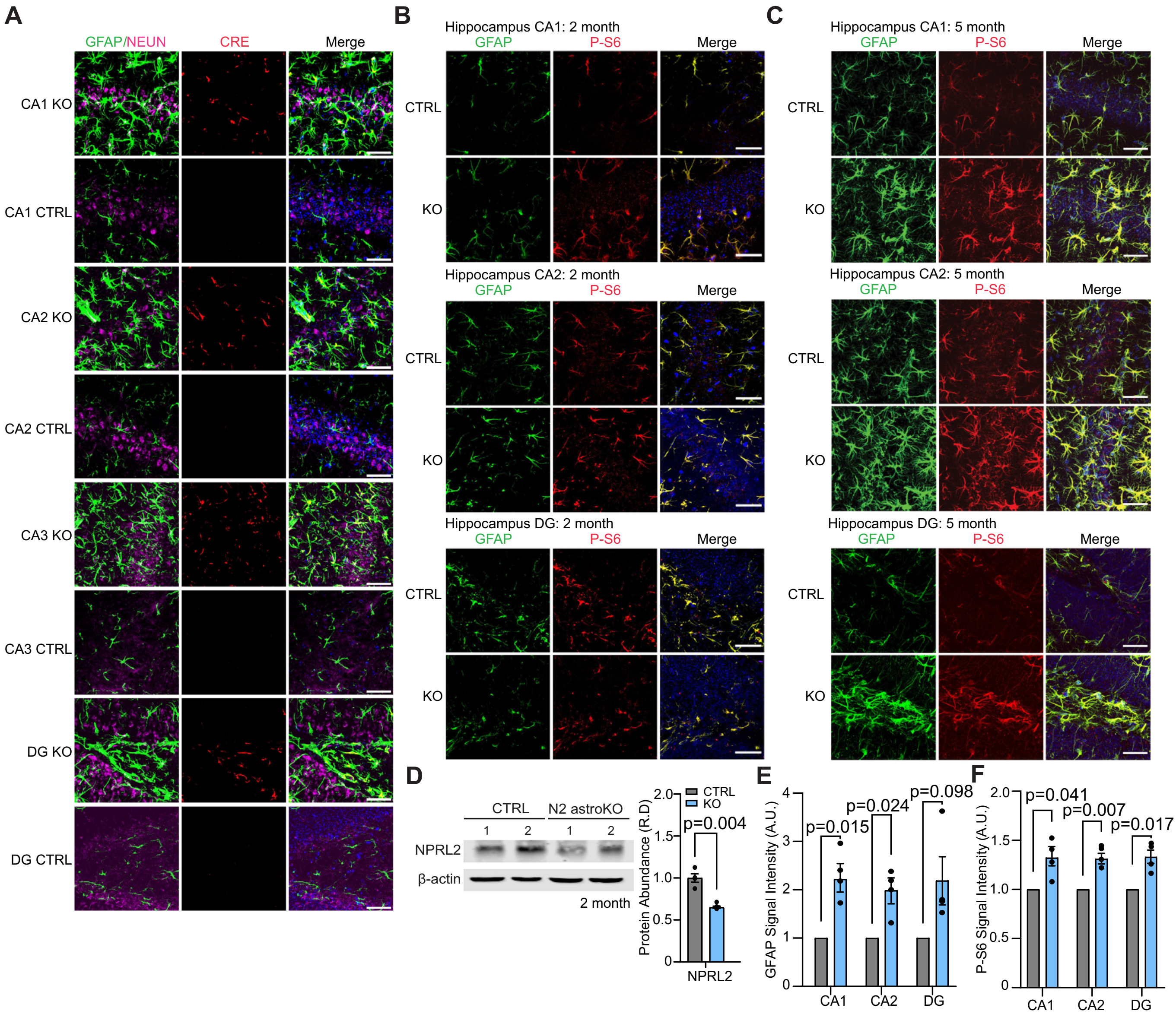

Figure EV1. GATOR1 deficiency sensitizes mice to age-dependent reactive astrocytosis.

(**A**) Representative confocal microscopy images of hippocampal CA1, CA2, CA3, and dentate gyrus (DG) sections at 5-month-old NPRL2 astroKO and control (CTRL) mice stained for GFAP, NEUN, CRE and DAPI. Scale bars = 40 µm. (**B**, **C**) Representative confocal microscopy images of hippocampal CA1, CA2, and DG regions from NPRL2 astroKO and control mice at (**B**) 2 months and (**C**) 5 months of age, stained for GFAP, P-S6 and DAPI. Scale bars = 40 µm. (**D**) Representative western blot analysis of NPRL2 and β-actin from NPRL2 astroKO and control brain protein extracts at 2 months of age. Quantification of NPRL2 abundance relative to β-actin is shown as relative density (R.D.) normalized to β-actin and includes all animals analyzed across two independent biological experiments ($n = 4$ mice per genotype). Welch's *t*-test was used for comparisons. (**E**, **F**) Quantification of average (**E**) GFAP and (**F**) P-S6 signal intensity in hippocampal CA1, CA2, and DG regions of control and NPRL2 astroKO mice at 5 months of age ($n = 4$ independent biological experiments; values represent KO normalized to matched WT within each experiment; two-tailed one-sample *t*-test vs 1 AU, arbitrary units). Data were shown as the mean ± SEM.

 © The Author(s)

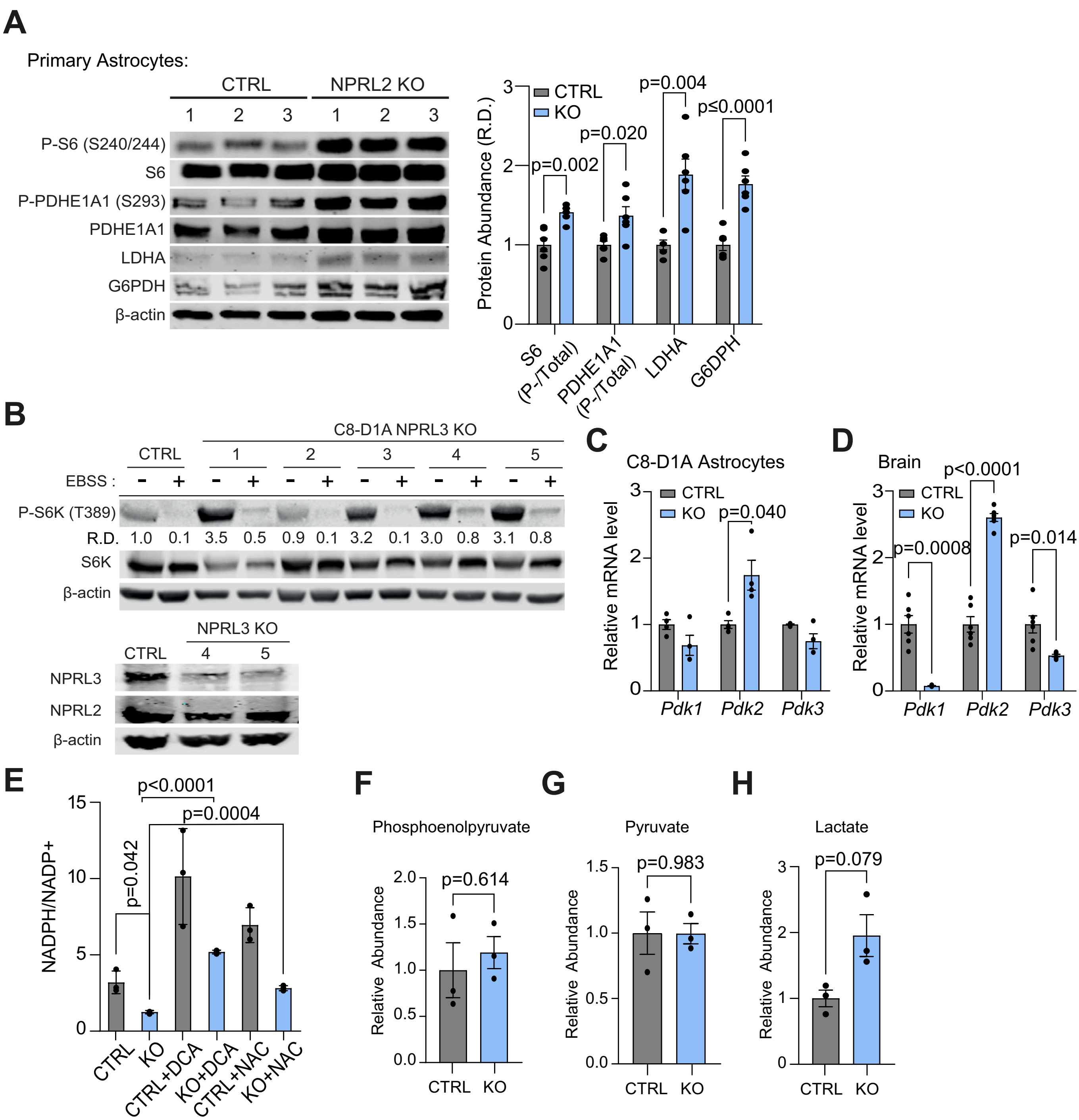

Figure EV2. Glycolysis and TCA cycle metabolic changes in GATOR1-deficient cells.

(**A**) Western blot analysis of P-S6 (S240/244), S6, P-PDHE1A1 (S293), PDHE1A1, LDHA, G6PDH, and β-actin of protein extracts from control (CTRL) and NPRL2 KO primary astrocytes ($n = 6$ independent cultures per genotype). Quantification of protein abundance is shown as relative density (RD) normalized to β-actin. Each data point represents an independent biological culture. (**B**) Western blot analysis of P-S6K (T389), S6K, and β-actin from parental control and N3KO C8-D1A cell lines treated with EBSS for 1 h. Quantification of P-S6K (T389) normalized to S6K and β-actin. NPRL3, NPRL2, and β-actin western blots from selected lines are shown for validation. (**C**) QRT-PCR analysis of pyruvate dehydrogenase kinase isoforms (*Pdk1*, *Pdk2*, and *Pdk3*) in N3KO and control C8-D1A cells ($n = 4$ independent cultures per genotype). (**D**) QRT-PCR analysis of pyruvate dehydrogenase kinase isoforms (*Pdk1*, *Pdk2*, and *Pdk3*) from brain extracts of mice NPRL2 astroKO and CTRL at 5 months ($n = 6$ mice per genotype; biological replicates). (**E**) Quantification of NADPH/NADP+ ratio in CTRL and N3KO C8-D1A cells treated with or without dichloroacetate (DCA; 5 mM) or *n*-acetyl-L-cysteine (NAC; 5 mM) for 1 h. Each data point represents an independent biological culture ($n = 3$ cultures per genotype). (**F–H**) Representative metabolite profiling of N3KO and control C8-D1A cells, including: (**F**) phosphoenolpyruvate, (**G**) pyruvate, and (**H**) lactate ($n = 3$ independent cultures per genotype). All mass spectrometry was replicated at least two times in independent experiments. Data were shown as the mean ± SEM. Welch's *t*-test and one-way ANOVA with post hoc Tukey were used for comparisons.

© The Author(s)

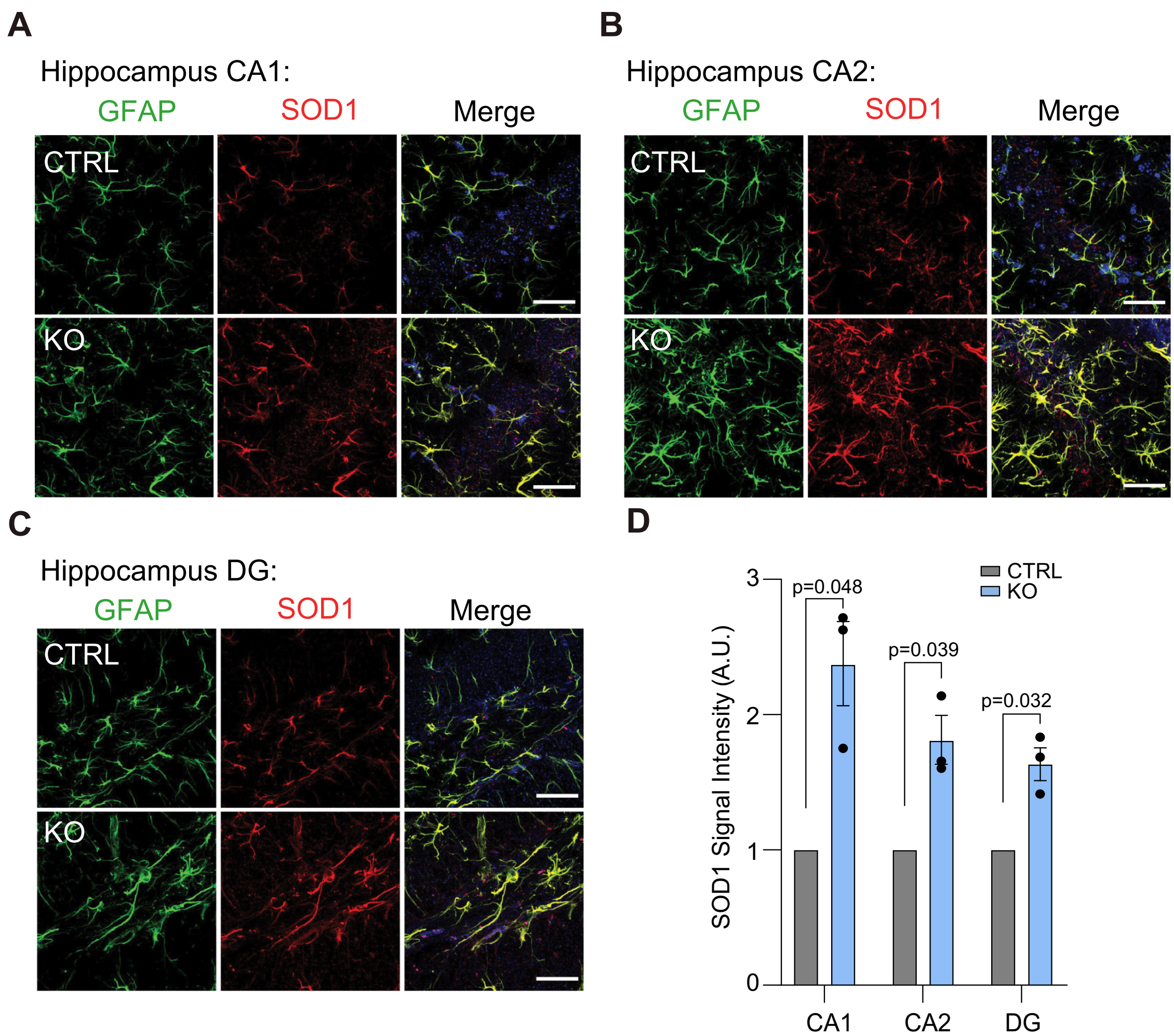

Figure EV3. Astrocytic GATOR1-deficiency increases hippocampal SOD1 expression.

(**A–C**) Representative confocal microscopy images of the hippocampal (**A**) CA1, (**B**) CA2, and (**C**) dentate gyrus (DG) regions stained for GFAP, SOD1, and DAPI. Scale bars = 40 µm. (**D**) Quantification of SOD1 signal intensity (arbitrary units, AU) in the indicated hippocampal regions. ($n = 3$ independent biological experiments; values represent KO normalized to matched WT within each experiment; two-tailed one-sample *t*-test vs 1 AU, arbitrary units). Data were shown as the mean ± SEM.

 © The Author(s)

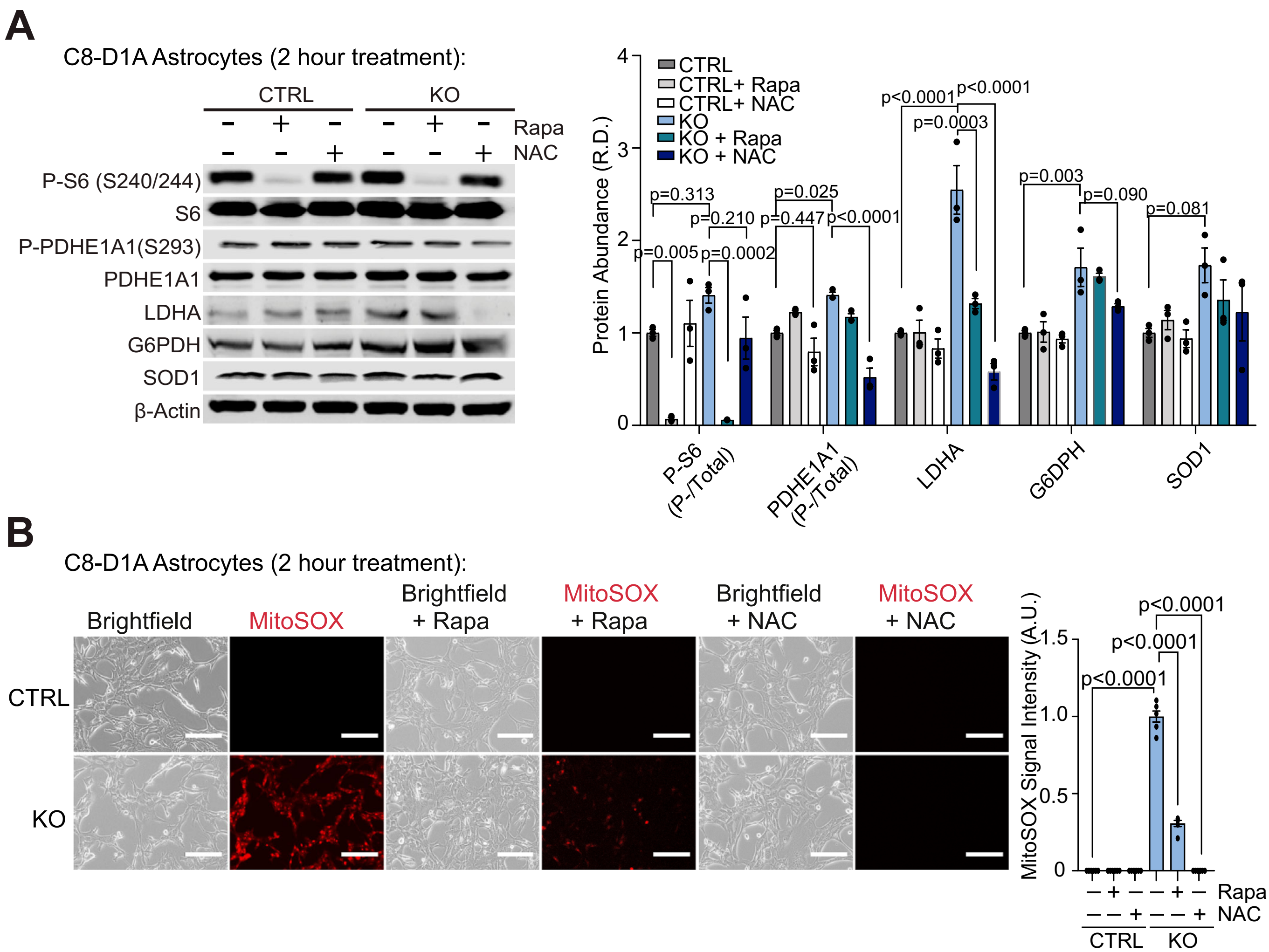

Figure EV4. Rapamycin and antioxidant treatment ameliorate GATOR1-dependent ROS.

(**A**) Representative western blot analysis of P-S6 (S240/244), S6, P-PDHE1A1 (S293), PDHE1A1, LDHA, G6PDH, SOD1, and β-actin from control (CTRL) and N3KO C8-D1A cells treated for 2 h with rapamycin (10 nM) or *n*-acetyl-L-cysteine (NAC; 5 mM) ($n = 3$ independent cultures per condition). Quantification of protein abundance is shown as relative density (R.D.), with phospho-protein levels standardized to their respective total protein levels and then normalized to β-actin. (**B**) Representative fluorescence images of CTRL and N3KO C8-D1A cells stained with MitoSOX Red for 15 min following pretreatment with rapamycin (10 nM) or NAC (5 mM) for 2 h, with quantification of fluorescent signal intensity (AU, arbitrary units). Each data point represents an independent biological culture ($n = 6$ cultures per genotype; biological replicates). Scale bar = 125 μm. Data were shown as mean ± SEM. One-way ANOVA with post hoc Tukey were used for comparison.

© The Author(s)

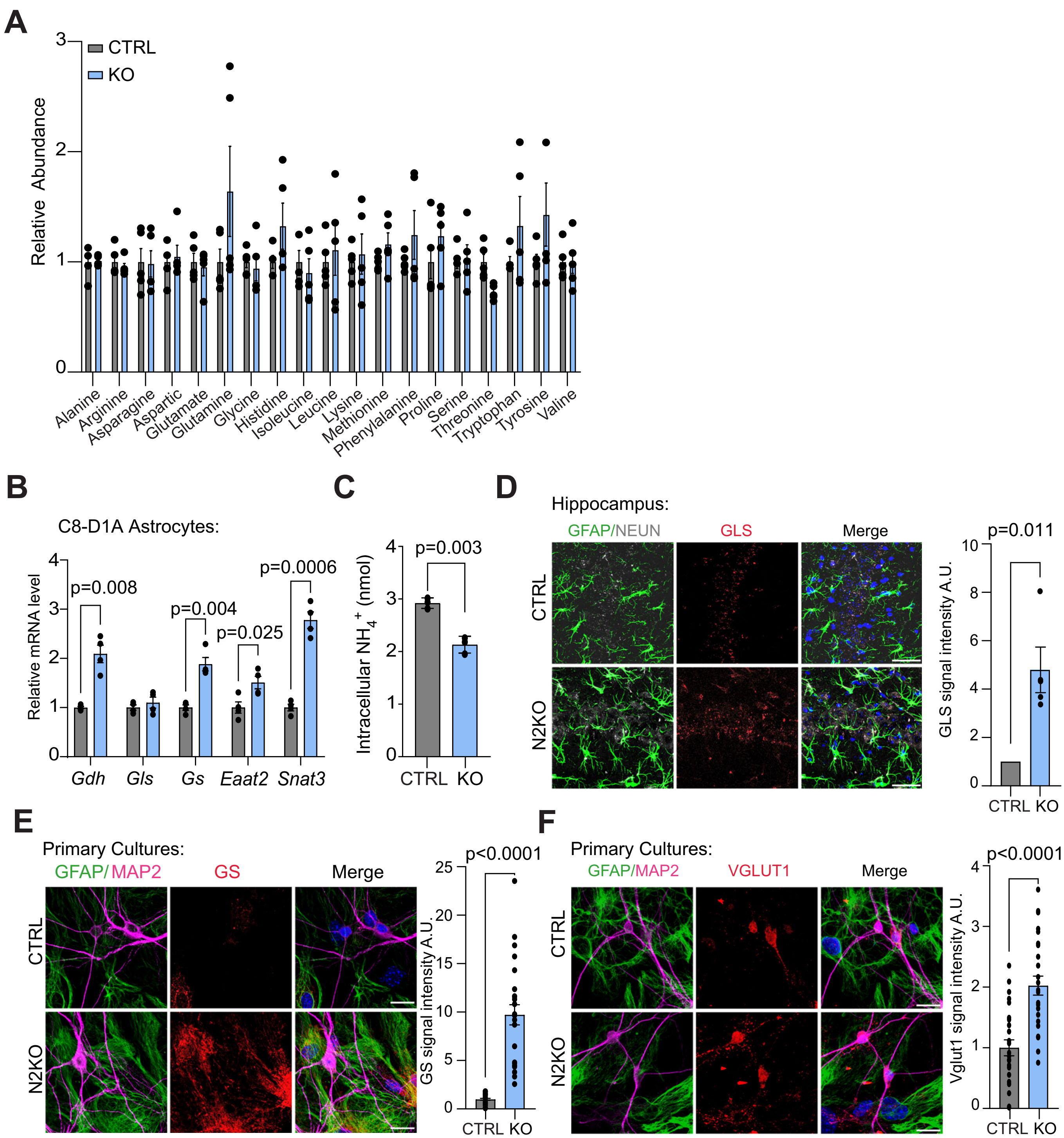

Figure EV5. GATOR1 defective astrocytes stimulate neuronal gene expression.

(**A**) Metabolomic analysis of relative amino acid abundance in brain extracts from NPRL2 astroKO and control (CTRL) mice at 2 months of age ($n = 5$ mice per genotype; biological replicates). (**B**) QRT-PCR analysis of metabolic enzymes (*Gdh*, *Gls,* and *Gs*) and astrocytic amino acid transporters (*Eaat2* and *Snat3*) from CTRL and N3KO C8-D1A cells ($n = 4$ cultures, independent cultures per genotype). (**C**) Ammonia (NH4+) concentration measured in CTRL and N3KO C8-D1A cells ($n = 3$ independent cultures per genotype). (**D**) Representative confocal microscopy images from 5-month-old CTRL and NPRL2 astrocKO mice stained for GFAP, NEUN, glutaminase (GLS), and DAPI. Quantification of GLS signaling intensity relative to CTRL is shown ($n = 5$ independent biological experiments; values represent KO normalized to matched WT within each experiment; two-tailed one-sample *t*-test vs 1 AU, arbitrary units). Scale bar = 40 µm. (**E**) Representative confocal microscopy images of co-cultures of control neurons with CTRL or NPRL2 KO primary astrocytes, stained for GFAP, MAP2, GS, and DAPI, with quantification of GS signal intensity in astrocytes ($n = 5$ individual cultures per genotype; 5 fields quantified per culture). Scale bar = 20 µm. (**F**) Representative confocal microscopy images of control neurons with CTRL or NPRL2 KO primary astrocytes, stained for GFAP, MAP2, VGLUT1, and DAPI. Quantification of vesicular glutamate transporter (VGLUT1) signal intensity in neurons ($n = 4$ individual cultures per genotype; 5 fields quantified per culture). Scale bar = 20 µm. Data were shown as the mean ± SEM. Welch's *t*-test was used for statistical comparisons.

 © The Author(s)
