# Supplementary material for: GATOR1 signaling defects promote astrocytic metabolic rewiring and excitatory neurotransmitter cycling
Source: EMBO Rep. 2026 Jul 3;27(15):4252–71. doi: 10.1038/s44319-026-00846-w (PMC13458053; doi:10.1038/s44319-026-00846-w)
Supplement: Supplementary file 2 [file 44319_2026_846_MOESM1_ESM.pdf]

EMBO reports

GATOR1 Signaling Defects Promote Astrocytic Metabolic Rewiring and Excitatory Neurotransmitter Cycling

Imane Hadj-Aissa, Maéline Muller, Jorge Soliz, Chantelle Sephton, and Paul Dutchak

Corresponding author(s): Paul Dutchak (paul.dutchak.1@ulaval.ca)

Review Timeline:

Submission Date:	1st Oct 25
Editorial Decision:	4th Nov 25
Revision Received:	21st Dec 25
Editorial Decision:	26th Jan 26
Revision Received:	27th Apr 26
Referee 1's comments on the revised manuscript	20th May 26
Advisor's Comments Received:	22nd May 26
Author's Response to the Advisor:	8th Jun 26
Accepted:	10th Jun 26

Editor: Esther Schnapp

Transaction Report:

(Note: With the exception of the correction of typographical or spelling errors that could be a source of ambiguity, letters and reports are not edited. Depending on transfer agreements, referee reports obtained elsewhere may or may not be included in this compilation. Referee reports are anonymous unless the Referee chooses to sign their reports.)

Dear Dr. Dutchak,

Thank you for the submission of your manuscript to EMBO reports. We have now received the full set of referee reports that is pasted below.

As you will see, the referees acknowledge that the findings are potentially interesting. However, they also have several suggestions for how the study could be improved and strengthened. I think all suggestions are good and should be addressed but please let me know in case you disagree and we can discuss the exact revision requirements further, also in a video chat, if you like.

I would thus like to invite you to revise your manuscript with the understanding that the referee concerns must be fully addressed and their suggestions taken on board. Please address all referee concerns in a complete point-by-point response. Acceptance of the manuscript will depend on a positive outcome of a second round of review. It is EMBO reports policy to allow a single round of major revision only and acceptance or rejection of the manuscript will therefore depend on the completeness of your responses included in the next, final version of the manuscript.

We realize that it is difficult to revise to a specific deadline. In the interest of protecting the conceptual advance provided by the work, we recommend a revision within 3 months (4th Feb 2026). Please discuss the revision progress ahead of this time with the editor if you require more time to complete the revisions.

You can either publish the study as a short report or as a full article. For short reports, the revised manuscript should not exceed 27,000 characters (including spaces but excluding materials & methods and references) and 5 main plus 5 expanded view figures. The results and discussion sections must further be combined, which will help to shorten the manuscript text by eliminating some redundancy that is inevitable when discussing the same experiments twice. For a normal article there are no length limitations, but it should have more than 5 main figures and the results and discussion sections must be separate. In both cases, the entire materials and methods must be included in the main manuscript file.

IMPORTANT NOTE: we perform an initial quality control of all revised manuscripts before re-review. Your manuscript will FAIL this control and the handling will be DELAYED if the following APPLIES:
1) A data availability section providing access to data deposited in public databases is missing. If you have not deposited any data, please add a sentence to the data availability section that explains that.
2) Your manuscript contains statistics and error bars based on n=2. Please use scatter blots in these cases. No statistics should be calculated if n=2.

When submitting your revised manuscript, please carefully review the instructions that follow below. Failure to include requested items will delay the evaluation of your revision.

1) a .docx formatted version of the manuscript text (including legends for main figures, EV figures and tables). Please make sure that the changes are highlighted to be clearly visible.

2) individual production quality figure files as .eps, .tif, .jpg (one file per figure). See https://wol-prod-cdn.literatumonline.com/pb-assets/embo-site/EMBOPress_Figure_Guidelines_061115-1561436025777.pdf for more info on how to prepare your figures.

3) We replaced Supplementary Information with Expanded View (EV) Figures and Tables that are collapsible/expandable online. A maximum of 5 EV Figures can be typeset. EV Figures should be cited as 'Figure EV1, Figure EV2" etc... in the text and their respective legends should be included in the main text after the legends of regular figures.

- For the figures that you do NOT wish to display as Expanded View figures, they should be bundled together with their legends in a single PDF file called *Appendix*, which should start with a short Table of Content. Appendix figures should be referred to in the main text as: "Appendix Figure S1, Appendix Figure S2" etc. See detailed instructions regarding expanded view here:

- Additional Tables/Datasets should be labeled and referred to as Table EV1, Dataset EV1, etc. Legends have to be provided in a separate tab in case of .xls files. Alternatively, the legend can be supplied as a separate text file (README) and zipped together with the Table/Dataset file.

4) a .docx formatted letter INCLUDING the reviewers' reports and your detailed point-by-point responses to their comments. As part of the EMBO Press transparent editorial process, the point-by-point response is part of the Review Process File (RPF), which will be published alongside your paper.

5) a complete author checklist, which you can download from our author guidelines . Please insert information in the checklist that is also reflected in the manuscript. The completed author checklist will also be part of the RPF.

6) Please note that all corresponding authors are required to supply an ORCID ID for their name upon submission of a revised manuscript (). Please find instructions on how to link your ORCID ID to your account in our manuscript tracking system in our Author guidelines

7) Before submitting your revision, primary datasets produced in this study need to be deposited in an appropriate public database (see https://www.embopress.org/page/journal/14693178/authorguide#datadeposition). Please remember to provide a reviewer password if the datasets are not yet public. The accession numbers and database should be listed in a formal "Data Availability" section placed after Materials & Method (see also https://www.embopress.org/page/journal/14693178/authorguide#datadeposition). Please note that the Data Availability Section is restricted to new primary data that are part of this study. * Note - All links should resolve to a page where the data can be accessed. *
If your study has not produced novel datasets, please mention this fact in the Data Availability Section.

8) At EMBO Press we ask authors to provide source data for the main manuscript figures. You will receive a separate email with instructions for providing source data with your revised manuscript, including how to upload and organize the files.

9) Our journal also encourages inclusion of *data citations in the reference list* to directly cite datasets that were re-used and obtained from public databases. Data citations in the article text are distinct from normal bibliographical citations and should directly link to the database records from which the data can be accessed. In the main text, data citations are formatted as follows: "Data ref: Smith et al, 2001" or "Data ref: NCBI Sequence Read Archive PRJNA342805, 2017". In the Reference list, data citations must be labeled with "[DATASET]". A data reference must provide the database name, accession number/identifiers and a resolvable link to the landing page from which the data can be accessed at the end of the reference. Further instructions are available at https://www.embopress.org/page/journal/14693178/authorguide#referencesformat

10) Regarding data quantification (see Figure Legends: https://www.embopress.org/page/journal/14693178/authorguide#figureformat)

The following points must be specified in each figure legend:

- the name of the statistical test used to generate error bars and P values,

- the number (n) of independent experiments (please specify technical or biological replicates) underlying each data point,

- the nature of the bars and error bars (s.d., s.e.m.),

- If the data are obtained from n {less than or equal to} 2, use scatter blots showing the individual data points.

Discussion of statistical methodology can be reported in the materials and methods section, but figure legends should contain a basic description of n, P and the test applied.

- Please also include scale bars in all microscopy images.

11) The journal requires a statement specifying whether or not authors have competing interests (defined as all potential or actual interests that could be perceived to influence the presentation or interpretation of an article). In case of competing interests, this must be specified in your disclosure statement. Further information: https://www.embopress.org/competing-interests

12) All Materials and Methods need to be described in the main text using our 'Structured Methods' format, which is required for all research articles. According to this format, the Methods section includes a separate Reagents and Tools Table file (listing key reagents, experimental models, software and relevant equipment and including their sources and relevant identifiers) and a Methods and Protocols section describing the methods using a step-by-step protocol format. The aim is to facilitate adoption of the methodologies across labs. More information on how to adhere to this format as well as a downloadable template (.docx) for the Reagents and Tools Table can be found in our author guidelines: https://www.embopress.org/page/journal/14693178/authorguide#structuredmethods.

An example of a Method paper with Structured Methods can be found here: https://www.embopress.org/doi/full/10.1038/s44320-024-00037-6#sec-4

We would also welcome the submission of cover suggestions, or motifs to be used by our Graphics Illustrator in designing a cover.

As part of the EMBO publication's Transparent Editorial Process, EMBO reports publishes online a Review Process File (RPF) to accompany accepted manuscripts. This File will be published in conjunction with your paper and will include the referee

reports, your point-by-point response and all pertinent correspondence relating to the manuscript.

You are able to opt out of this by letting the editorial office know (emboreports@embo.org). If you do opt out, the Review Process File link will point to the following statement: "No Review Process File is available with this article, as the authors have chosen not to make the review process public in this case."

I look forward to seeing a revised form of your manuscript when it is ready.

Best regards,
Esther

Esther Schnapp, PhD
Senior Editor
EMBO reports

Referee #1:

Hadj-Aissa et al. examined astrocyte-specific loss of GATOR1 (NPRL2/NPRL3) and claim it causes metabolic rewiring, complex II dysfunction, excess ROS, and secondary enhancement of the glutamate-glutamine cycle leading to seizures. The topic is interesting and I enjoyed reading the paper which and addresses an underexplored non-neuronal aspect of GATORopathies within the realm of mTORopathies.
The overall story is coherent, but several experimental elements do not yet convincingly support the conclusions. The link from astrocytic mTORC1 activation to neuronal excitatory imbalance is plausible but, in my view, only partially demonstrated. Some of the quantitative evidence (especially WB and qPCR) is based on small n (mostly n = 3-4), and effect sizes are modest-often 20-40 % raising concerns about robustness. Statistical tests are all uncorrecteed pairwise t-tests. Several conclusions depend on western blots or immunostaining without clear quantification.

Comments

1) Western blot data (Fig 1A-B, Fig 2A. Bands are few and sometimes faint; n = 3-4 is low, and fold-changes in phospho-S6 0r PDHE1A1 are small (<1.5×). These differences might reflect variability rather than genuine pathway activation. Loading control is highly variable.

2)The authors mention that GFAP-Cre system can recombine in some neuronal precursors. Staining (GFAP/NeuN) would strengthen conclusions. The seizure data are descriptive; ephys recordings would confirm network hyperexcitability.

3) Authors report reduced CII-dependent respiration, but n = 3 and SEM are overlaping, thus limit confidence. DCA "rescue" is partial and shold be statistically compared. ROS quantification relies on MitoSOX fluorescence (qualitative) flow or plate-based readouts be more reliable.

4) Increases in glutamine/lactate are small and from n = 3 replicates . No isotope-tracing or flux analysis is presented, so "enhanced glutamine synthesis" remains inferential. Likewise, the in-vivo p180 assay shows only a trend, not a significant change.

5) The co-culture experiment suggests increased neuronal VGLUT1 when grown on KO astrocytes, but causality between astrocytic metabolic output and neuronal phenotype is not tested or established (e.g. no rescue, no conditioned-medium swap). Hence, the claim that astrocytic GATOR1 "drives excitatory neurotransmitter cycling" feels overinterpretation

6) The idea that astrocytic mTORC1 deregulation affects neuronal excitability is interesting but not entirely new; similar conclusions were reported in TSC1-GFAP KO models. Novelty is the link it to Complex II defects, but still this part remains speculation.

Referee #2:

Hadj-Aissa et al. generate an asotrocyte-specific knockout of Nprl2, a subunit of the mTORC1-regulating GATOR1 complex. Using a combination of in vitro and in vivo experiments, the authors characterize the consequences for cellular metabolism, neurotransmitter excretion and brain physiology. This is an interesting and relevant paper that yields novel insights into cell-type specific functions of GATOR1, with implications for physiological roles of the mTORC1 pathway and pathogenic GATOR1 mutations associated with neurological disorders. The manuscript is easy to follow; the data are clearly presented and of overall high quality. I only have a few suggestions that might help the authors to support the chain of causality of their model.

Major points:

1. Does mTORC1 inhibition alleviate the phenotypes of cultured Nprl2 KO astrocytes? I don't think this has to be investigated for all phenotypes, but a few examples would be helpful to directly connect the loss of Nprl2 to mTORC1 and its therapeutic potential.

2. Along the same lines, do antioxidants suppress the metabolite or gene expression changes observed in Nprl2 KO astrocytes? Again, an exhaustive analysis might be more suitable for follow-up studies, but some proof-of-principle would provide support for the author's model that elevated ROS generation instruct the metabolic reprogramming they observe.

Minor points:

1. Fig. EV3B: Please also include Nprl2 to confirm loss of the protein in the CRISPR KO cells.

2. Fig. EV3B: The authors conclude that Nprl2-deficient cells have impaired mTORC1 inactivation upon starvation. I agree that p-S6K is higher in KO cells than controls upon EBSS treatment, but they still show a strong nutrient response. As the KO cells have a much higher basal p-S6K signal, the relative change might actually be comparable. Does this mean that astrocyte mTORC1 is less reliant on GATOR1 to sense amino acids?

Referee #3:

The manuscript by Hadj-Aissa et al, entitled 'GATOR1 signalling defects drive astrocytic metabolic rewiring and promote excitatory neurotransmitter cycling' uses in vitro and in vivo models to investigate the effects of astrocytic-specific KO of the mTORC1 negative regulator, GATOR1. This is an interesting and timely study; as the author point out, there is growing interest in GATOR1 mutations as an underlying cause of epilepsies but much of the data is focused on neuron cell-specific roles. Astrocytes play and important regulatory and protective role in the brain and can directly influence neuronal function.

The data are well presented, with appropriate repeats and statistical analysis. Overall, I think the findings are very interesting and raise a lot of questions that will reveal new insights into the underlying mechanisms of epilepsy. As such, I think it is an important addition to the field. The astrocyte data is fairly robust but the latter data, looking at potential astrocyte impact on neurons is very correlative at this point. I don't think this is a problem for the manuscript per se, but the authors should tone down their conclusions. A lot of in vitro and in vivo work would need to be done to validate their model. The authors should instead concentrate on the metabolic rewiring of astrocytes they have observed. There are several comments below that would strengthen the conclusions of the paper:

The main points

• It is important to show that the key phenotypes in Fig.2 /3 are definitely induced downstream of mTORC1 activation by treatment with rapamycin or rescue NPLR3 expression (in vitro cell culture only).
• Fig.1I: From 2 months to 5months, are there more astrocytes in the controls or is there a natural increase in reactivity? i.e the GFAP signal is generally much stronger at 5months. Are the astrocytes dividing? And if so, could the difference be mTORC1-driven cell cycle differences? Or perhaps there are natural changes in astrocyte metabolism with age?
• Along the same lines, can you add more discussion about what might be happening between 2months and 5 months that leads to the cellular dysfunction - is an accumulation of damage? How quickly after the KO of NPLR3 in cells do you see metabolic changes?
• Why did you generate NPLR3 KO in the C8-D1A cells instead of NPLR2? Do you have any evidence that they phenocopy exactly?
• In the discussion, the authors suggest there may be impaired SDH activity which is underlying the metabolic rewiring- can the authors test this?

Minor comments:

• Include a clearer blot for NPRL2 and pS6K(T389) in Fig.1A
• Typo on page 5: reference to Fig.1E-1G should be 1F-1G.
• Fig.EV3B isn't very convincing. Can you quantify the signal remaining in KOs upon HBSS treatment?
• Can you explain why you would see a decrease in pdk1 and 3, while an increase in pdk2?
• Although not significant, Complex I does seem to be reduced in KOs in EV5C - can the authors comment on this?
• Could you clarify in the text, on page 7 ' DCA treatment...rescued CII activity to control levels' are you referring to KO+DCA compared to KO control?
• Can you quantify Fig.3D
• Why did you decide to only look at SOD mRNA levels? Do you have any explanation for why SOD3 expression is decreased.
• In several figures and panels, the authors refer to 'neighbouring cells' either in relation to astrocytes or neurons. Where this is the case, could the authors show a high-resolution zoom of the images in question and use arrows to highlight exactly what they are referring to?
• Is there any evidence, e.g. by blue native PAGE that there are defects in the mitochondrial complexes CII and CIV?

- Can you add an explanation of why you did metabolomics on the 2month old mice, before obvious changes/phenotypes are observed?
- For completeness, it would be worth adding the qPCR data for GLS and GDH to Fig.EV6C
- Can the authors discuss why Gdh expression shows a significant increase in cells (but decrease in tissues) in Fig.4A/EV6B?
- Can the authors comment on why astrocytic glutamate transporters might be increased in GATOR1 KO? Does this suggest that they are both producing more glutamate and taking more up from the environment.

Referee #1:

Hadj-Aissa et al. examined astrocyte-specific loss of GATOR1 (NPRL2/NPRL3) and claim it causes metabolic rewiring, complex II dysfunction, excess ROS, and secondary enhancement of the glutamate-glutamine cycle leading to seizures. The topic is interesting and I enjoyed reading the paper which and addresses an underexplored non-neuronal aspect of GATORopathies within the realm of mTORopathies.
The overall story is coherent, but several experimental elements do not yet convincingly support the conclusions. The link from astrocytic mTORC1 activation to neuronal excitatory imbalance is plausible but, in my view, only partially demonstrated. Some of the quantitative evidence (especially WB and qPCR) is based on small n (mostly n = 3-4), and effect sizes are modest-often 20-40 % raising concerns about robustness. Statistical tests are all uncorrecteed pairwise t-tests. Several conclusions depend on western blots or immunostaining without clear quantification.

Comments
1) Western blot data (Fig 1A-B, Fig 2A. Bands are few and sometimes faint; n = 3-4 is low, and fold-changes in phospho-S6 0r PDHE1A1 are small (<1.5×). These differences might reflect variability rather than genuine pathway activation. Loading control is highly variable.

We thank the reviewer for this point. All western blotting in this study was performed using fluorescence-based LI-COR imaging, which has a very broad linear dynamic range and low background, permitting accurate quantification even for targets with modest fold-changes; performance that is superior to chemiluminescent detection. As clarified in the revised Methods, phospho-signals were quantified as Phospho/Total ratios (normalized to β-actin), reducing variability introduced by loading differences. While the absolute fold-changes in P-S6 and P-PDHE1A1 are moderate (1.5-fold), they are directionally consistent across samples, statistically significant and concordant with multiple readouts of mTORC1 activation.

2)The authors mention that GFAP-Cre system can recombine in some neuronal precursors. Staining (GFAP/NeuN) would strengthen conclusions. The seizure data are descriptive; ephys recordings would confirm network hyperexcitability.

We appreciate this thoughtful comment. We note that the GFAP-IRES-Cre line used here is substantially more astrocyte-restricted than the older hGFAP-Cre (25MES driver) lines known for broader recombination. Although validated IHC-grade antibodies for GATOR1 subunits, including NPRL2 and NPRL3, are not available, we performed CRE immunostaining, which shows clear colocalization with GFAP+ in our

model, supporting astrocyte-specific recombination (Figure EV1A). To address potential neuron-intrinsic contributions, we complemented *in vivo* experiments with astrocyte-neuron co-culture studies. Neurons were wild-type, whereas only astrocytes carried the NPRL2 deletions, yet neurons still acquired the metabolic/VGLUT1 phenotype. This strongly supports an astrocyte-autonomous mechanism rather than recombination in neuronal precursors.

Regarding seizures, our intent in this short-format report is to establish the metabolic mechanism and the underlying astrocyte-dependent pathology. We therefore document convulsive events (Video EV1, Figure 1E). We agree that *in vivo* electrophysiological recordings would add valuable regional resolution, however, such experiments fall beyond the scope of this present mechanistic study and represent an important future direction.

3) Authors report reduced CII-dependent respiration, but n = 3 and SEM are overlaping, thus limit confidence. DCA "rescue" is partial and shold be statistically compared. ROS quantification relies on MitoSOX fluorescence (qualitative) flow or plate-based readouts be more reliable.

We appreciate the careful examination of the respirometry dataset. The SEM overlap referenced appears primarily at Complex I, where we indeed observed no genotype-specific effect, consistent with a selective Complex II defect. In contrast, Complex II-supported respiration is robustly and significantly reduced, and Complex IV activity shows a secondary decrease expected when upstream Complex II delivery is impaired. We now explicitly state that each n=3 represents distinct, independently prepared biological replicates, which are run on separate days, not technical replicates from the same experiment.

DCA acts upstream of these complexes via PDH activation (PDK inhibition), and is not expected to fully normalize electron transport chain defects. The observed partial rescue aligns with known metabolic positioning of PDH relative to ETC function. Statistical comparisons for DCA effects have now been added.

MitoSOX staining is a reliable method to determine reactive oxygen species formation from the mitochondria as used in multiple publications (doi: 10.1016/j.bbrc.2007.04.106). Our imaging data reflect a large and reproducible genotype-dependent different, with controls consistently at or near-baseline intensity. While flow-based or plate-reader quantification can add additional layers of resolution, the magnitude and consistency of our results are strong and clear.

4) Increases in glutamine/lactate are small and from n = 3 replicates . No isotope-tracing or flux analysis is presented, so "enhanced glutamine synthesis" remains inferential. Likewise, the in-vivo p180 assay shows only a trend, not a significant change.

We agree that isotope tracing would establish metabolic flux directly. Accordingly, we have refined our language to state that GATOR1 deficiency enhances the GS pathway, with supporting data showing more glutamine abundance, rather than proving increased flux *per se*. We state that extracellular glutamine and lactate increases are statistically significant, derived from 3 independent biological replicates with replicated data obtained in two different mass spectrometer experiments. These metabolite rises align with: 1) GS upregulation *in vitro* and *in vivo*, 2) reduced intracellular NH+4, consistent with GS activity, 3) upregulation of EAAT1/2 and SNAT3, indicating enhanced glutamate uptake and glutamine export. While we do not assert direction flux increases, the convergence of enzyme levels, intracellular metabolic changes, transporter upregulation, and extracellular metabolic levels support enhanced pathway engagement. Regarding the p180 datases, we agree the 2-month glutamine increase does not reach significance. We interpret the directional trend as evidence of early compensatory buffering in intact brain prior to seizure onset. Later pathological stages likely reveal the phenotype more strongly. Metabolite tracing studies are an important future direction but lie beyond the scope of this concise report.

5) The co-culture experiment suggests increased neuronal VGLUT1 when grown on KO astrocytes, but causality between astrocytic metabolic output and neuronal phenotype is not tested or established (e.g. no rescue, no conditioned-medium swap). Hence, the claim that astrocytic GATOR1 "drives excitatory neurotransmitter cycling" feels overinterpretation

We thank the reviewer for raising this fair point. We have now softened the claim from
“drives excitatory neurotransmitter cycling” to “promotes excitatory neurotransmitter cycling.” The co-culture experiment demonstrates sufficiency, wild-type neurons exposed only to NPRL2 KO astrocytes upregulate VGLUT1, consistent across replicates. This neuronal phenotype aligns with the noted astrocytic change, and neuronal SNAT and GLS upregulation. Together, these findings support an astrocyte-to-neuron glutamine supply mechanism within our model.

6) The idea that astrocytic mTORC1 deregulation affects neuronal excitability is interesting but not entirely new; similar conclusions were reported in TSC1-GFAP KO models. Novelty is the link it to Complex II defects, but still this part remains speculation.

We appreciate this insightful comment. Prior studies, including the TSC1-GFAP KO models, support mTORC1 influences neuronal excitability. Our work advances this concept in two distinct ways. First, no previous study has identified GATOR1, an intracellular amino-acid signaling complex, as the regulator of astrocytic mTORC1 driving circuit. Secondly, the downstream mechanism of GATOR1 selectively effecting Complex II supported respiration defects, SDHA transcriptional downregulation, increased ROS, reduced NAPDH, and resulting enhancement of GS/glutamine pathway. This is strengthened by the new data we include showing SDHA downregulation, a core CII-subunit, in GATOR1-deficient astrocytes. This establishes a clear mechanistic bridge from amino-acid signaling to ETC function to neuronal support. While additional studies could deepen the circuit-level implications, we believe this mechanistic axis constitutes a substantial conceptual advancement beyond existing mTORC1 astrocyte literature.

Referee #2:

Hadj-Aissa et al. generate an asotrocyte-specific knockout of Nprl2, a subunit of the mTORC1-regulating GATOR1 complex. Using a combination of in vitro and in vivo experiments, the authors characterize the consequences for cellular metabolism, neurotransmitter excretion and brain physiology. This is an interesting and relevant paper that yields novel insights into cell-type specific functions of GATOR1, with implications for physiological roles of the mTORC1 pathway and pathogenic GATOR1 mutations associated with neurological disorders. The manuscript is easy to follow; the data are clearly presented and of overall high quality. I only have a few suggestions that might help the authors to support the chain of causality of their model.

Major points:

1. Does mTORC1 inhibition alleviate the phenotypes of cultured Nprl2 KO astrocytes? I don't think this has to be investigated for all phenotypes, but a few examples would be helpful to directly connect the loss of Nprl2 to mTORC1 and its therapeutic potential.

We thank the reviewer for this important suggestion. Numerous studies have demonstrated that mTORC1 inhibition by rapamycin or rapalogs alleviate phenotypes driven by loss of upstream negative regulators (ex. TSC1, TSC2, DEPDC5) (doi: 10.1002/gcc.20118; doi: 10.1002/ana.20784; PMID: 18389497; DOI: 10.1093/hmg/ddz123). While a full therapeutic characterization is beyond the scope of this short-format mechanistic report, we now provide new experimental evidence directly addressing this point. Specifically, we show that 24 hour rapamycin treatment of the astrocytes robustly suppresses P-S6 (mTORC1 activity), normalizes LDHA and G6PHD expression, decreases SOD1, and fully suppresses elevated mitochondrial ROS levels (Fig 4). These results demonstrate that mTORC1 hyperactivity contributes to several components of the metabolic redox phenotype. We have integrated these findings, emphasizing the therapeutic relevance while noting that more extensive rescue studies will be a focus of follow-up work.

2. Along the same lines, do antioxidants suppress the metabolite or gene expression changes observed in Nprl2 KO astrocytes? Again, an exhaustive analysis might be more suitable for follow-up studies, but some proof-of-principle would provide support for the author's model that elevated ROS generation instruct the metabolic reprogramming they observe.

We thank the reviewer for this insightful suggestion. We now included new antioxidant treatment data demonstrating that N-acetyl-l-cysteine (NAC), fully rescues ROS levels within 2 hours, reduces the inhibitory P-PDHE1A1, normalizes LDHA and reduces G6PHD in the KO astrocytes. These findings (Fig 4, EV4) provide direct support for our model that ROS is not merely a consequence of the ETC dysfunction but contributes to shaping the metabolic reprogramming phenotype.

Minor points:

1. Fig. EV3B: Please also include Nprl2 to confirm loss of the protein in the CRISPR KO cells.

We thank the reviewer for this suggestion. We have now included additional western blots demonstrating NPRL3 deletion does not alter NPRL2 abundance, confirming specificity in the CRISPR approach. These data are now incorporated into Figure EV2B.

2. Fig. EV3B: The authors conclude that Nprl2-deficient cells have impaired mTORC1 inactivation upon starvation. I agree that p-S6K is higher in KO cells than controls upon EBSS treatment, but they still show a strong nutrient response. As the KO cells have a much higher basal p-S6K signal, the relative change might actually be comparable. Does this mean that astrocyte mTORC1 is less reliant on GATOR1 to sense amino acids?

We thank to reviewer for raising this question. We have revised the text to clarify two key points. First, EBSS removes both amino acids and growth factors, providing a strong stimulus for mTORC1 inhibition through both GATOR1- and TSC-dependent pathways. Secondly, the upon quantification of P-S6K/S6K ratios, KO cells indeed exhibit a decrease, but the basal hyperphosphorylation remains substantially higher than controls (~9-fold), resulting in a persistently elevated mTORC1 setpoint. Taken together, these observations support that both GATOR1 and TSC pathways cooperate to fully suppress mTORC1 in astrocytes. Therefore, we do not interpret these data to mean that astrocytic mTORC1 is less reliant on GATOR1, but rather the loss of GATOR1 removes only one arm of a dual regulatory system, which has important implications for interpreting older TSC-focused starvation studies.

Referee #3:

The manuscript by Hadj-Aissa et al, entitled 'GATOR1 signalling defects drive astrocytic metabolic rewiring and promote excitatory neurotransmitter cycling' uses in vitro and in vivo models to investigate the effects of astrocytic-specific KO of the mTORC1 negative regulator, GATOR1. This is an interesting and timely study; as the author point out, there is growing interest in GATOR1 mutations as an underlying cause of epilepsies but much of the data is focused on neuron cell-specific roles. Astrocytes play and important regulatory and protective role in the brain and can directly influence neuronal function.

The data are well presented, with appropriate repeats and statistical analysis. Overall, I think the findings are very interesting and raise a lot of questions that will reveal new insights into the underlying mechanisms of epilepsy. As such, I think it is an important addition to the field. The astrocyte data is fairly robust but the latter data, looking at potential astrocyte impact on neurons is very correlative at this point. I don't think this is a problem for the manuscript per se, but the authors should tone down their conclusions. A lot of in vitro and in vivo work would need to be done to validate their model. The authors should instead concentrate on the metabolic rewiring of astrocytes they have observed. There are several comments below that would strengthen the conclusions of the paper:

The main points

• It is important to show that the key phenotypes in Fig.2 /3 are definitely induced downstream of mTORC1 activation by treatment with rapamycin or rescue NPLR3 expression (in vitro cell culture only).

We agree that linking the phenotypes directly to mTORC1 strengthens the mechanistic framework. We now include new rapamycin treatment data demonstrating that 24 hour mTORC1 inhibition represses P-S6, reduces LDHA and G6PDH, decreases SOD1 and fully normalizes ROS after prolonged exposure (Fig 4). These finding provide a clear functional link between mTORC1 hyperactivation, as a result of GATOR1 dysfunction, and multiple aspects of the metabolic phenotype. We also note the temporal limitations of rapamycin, acute inhibition can rapidly correct signaling abnormalities (eg. P-S6) whereas reversal of metabolic rewiring (PPP, PDH, ROS accumulation) has slower kinetics. This may explain why short-term rapamycin only partially reverses some metabolic parameters. Re-expression of GATOR1 subunits has been addressed in our prior KO characterization (Muller et al. 2024) where NPRL2 and NPRL3 deletion produced equivalent mTORC1 outcomes.

• Fig.1I: From 2 months to 5months, are there more astrocytes in the controls or is there a

natural increase in reactivity? i.e the GFAP signal is generally much stronger at 5months. Are the astrocytes dividing? And if so, could the difference be mTORC1-driven cell cycle differences? Or perhaps there are natural changes in astrocyte metabolism with age?

We thank the reviewer for raising this important point. We have now quantified the number of GFAP+ cells per field in 2- versus 5-month control and KO tissue, and confirm that cell number does not differ significantly between genotypes. A modest increase in astrocytes is observed with aging, but again this is independent of genotype. The changes in GFAP signal reflects morphological hypertrophy/reactivity of the astrocytes, not proliferation. GATOR1 loss therefore appears to potentiate age-associated reactive pathways beyond physiological levels.

• Along the same lines, can you add more discussion about what might be happening between 2months and 5 months that leads to the cellular dysfunction - is an accumulation of damage? How quickly after the KO of NPLR3 in cells do you see metabolic changes?

We now expand our explanation to address this temporal transition, supporting a two-phase model. We now emphasize that multiple processes likely act synergistically between 2 and 5 months to drive the transition from a mild biochemical abnormality to overt astrocytic dysfunction and seizure onset. GFAP morphology remains largely normal at 2 months, suggesting that early metabolic rewiring precedes structural pathology. We also highlight recent metabolomic studies showing age-dependent shifts in metabolism in mice, and humans, which may amplify the vulnerability created by GATOR1 loss, strengthening the late-onset phenotype. Seizure emergence itself is known to induce reactive astrocytes and metabolic remodeling, providing an additional feed-forward mechanism that may accelerate dysfunction after 5 months. Finally, we note that determining the precise onset of metabolic alterations in our CRISPR models is technically constrained by time for the generation, selection and expansion of CRISPR-edited clones, which requires multiple days of culture and making it impractical to resolve hour-scale metabolic transitions immediately following gene deletion. Together, these considerations support a model in which baseline GATOR1-dependent metabolic stress, age-related metabolic drift, and seizure-induced glial remodeling collectively drive the progressive phenotype observed in vivo.

• Why did you generate NPLR3 KO in the C8-D1A cells instead of NPLR2? Do you have any evidence that they phenocopy exactly?

We appreciate the opportunity to clarify this decision. NPRL2 and NPRL3 function together with DEPDC5 as obligate components of the GATOR1 complex. As shown

previously (Bar-Peled et al. 2013; Muller et al. 2024) loss of either NPRL2 or NPRL3 abolishes GATOR1 function, producing equivalent defects in mTORC1 regulation, starvation responses and amino acid signaling. In our hands, both NPRL2-KO and NPRL3-KO astrocytes display indistinguishable mTORC1 hyperactivity, P-PDH, PPP engagement and redox phenotypes, supporting the use of NPRL3 KO as a tractable *in vitro* model. We now also clarify this in the text.

• In the discussion, the authors suggest there may be impaired SDH activity which is underlying the metabolic rewiring- can the authors test this?

We thank the reviewer for this helpful suggestion. We now include new transcript-level data showing significant downregulation of SDHA, the catalytic core subunit of Complex II, in GATOR1-deficient astrocytes (Fig.3). This result directly supports our interpretation of impaired CII function and strengthens the mechanistic connection between GATOR1 loss, TCA cycle rerouting, and elevated ROS.

Minor comments:

• Include a clearer blot for NPRL2 and pS6K(T389) in Fig.1A

We have now provided a darker exposure of these western blot panels, and provide the full blot scans for editorial review.

• Typo on page 5: reference to Fig.1E-1G should be 1F-1G.

Corrected. Thank you.

• Fig.EV3B isn't very convincing. Can you quantify the signal remaining in KOs upon HBSS treatment?

We agree and now include the relative density values in the figure.

• Can you explain why you would see a decrease in pdk1 and 3, while an increase in pdk2?

We have added a short explanation that PDK isoforms are subject to distinct transcriptional control mechanisms in a tissue- and context-dependent manner. PDK2 has been reported to be preferentially regulated by nutrient- and growth-factor signaling in metabolically active cells, including astrocytes, whereas PDK1 and PDK3 are more strongly induced by hypoxia-responsive pathways. This divergence is

consistent with selective engagement of the mTORC1-PDH regulatory axis in our model.

• Although not significant, Complex I does seem to be reduced in KOs in EV5C - can the authors comment on this?

We note that a modest Complex I reduction is observed, but it does not reach significance and is far smaller than the pronounced Complex II defect (see scale). We interpret CI difference as a secondary consequence of altered redox state rather than a primary defect.

• Could you clarify in the text, on page 7 ' DCA treatment...rescued CII activity to control levels' are you referring to KO+DCA compared to KO control?

Thank you for noting this point. We now state that KO+DCA is restored to the level of control + vehicle, not simply improved relative to KO.

• Can you quantify Fig.3D

We now include the quantification.

• Why did you decide to only look at SOD mRNA levels? Do you have any explanation for why SOD3 expression is decreased.

We focused on Sod1/2/3 because they represent the canonical mitochondrial and cytosolic superoxide dismutatses. Sod1 is selectively elevated in the KO cells, consistent with mitochondrial ROS spillover. Sod3, an extracellular isoform, shows reduced expression, which may reflect a shift from extracellular to intracellular antioxidant demand.

• In several figures and panels, the authors refer to 'neighbouring cells' either in relation to astrocytes or neurons. Where this is the case, could the authors show a high-resolution zoom of the images in question and use arrows to highlight exactly what they are referring to?

We now include high-magnification insets in Figure 3 to clearly show the neighbouring cells adjacent to GFAP+ astrocytes.

• Is there any evidence, e.g. by blue native PAGE that there are defects in the mitochondrial complexes CII and CIV?

While we do not include BN-PAGE, we now provide molecular evidence (reduced Sdha transcript expression) and functional ETC measurements (Oroboros assays) showing impaired CII and CIV activity. These complementary datasets demonstrate a coordinated mitochondrial dysfunction.

• Can you add an explanation of why you did metabolomics on the 2month old mice, before obvious changes/phenotypes are observed?

These metabolomic studies were designed as an unbiased early-timepoint screen to detect pre-symptomatic biochemical shifts before overt pathology. We clarify this rationale in the text.

• For completeness, it would be worth adding the qPCR data for GLS and GDH to Fig.EV6C

We now include the GLS and GDH QRT-PCR values.

• Can the authors discuss why Gdh expression shows a significant increase in cells (but decrease in tissues) in Fig.4A/EV6B?

The increased GDH expression in KO astrocytes is consistent with redox-driven metabolic compensation, whereas reduced GDH levels in whole-tissue extracts likely reflects the influence of non-astrocytic cell populations that are obscured in bulk tissue analysis.

• Can the authors comment on why astrocytic glutamate transporters might be increased in GATOR1 KO? Does this suggest that they are both producing more glutamate and taking more up from the environment.

We clarify that the elevated astrocytic EAAT1/2 expression likely represents a compensatory response to limit glutamate toxicity. In GATOR1-deficient astrocytes, increased glutamine synthesis is expected to elevate glutamate flux within the neuron-astrocyte cycle. Upregulation of these transporters would serve to enhance glutamate clearance, protect against excitotoxicity, and maintain neurotransmitter cycling efficiency, consistent with prior reports in hyperexcitable or seizure-prone networks. (DOI: 10.1016/s0301-0082(00)00067-8)

Dear Dr. Dutchak,

Thank you for the submission of your revised manuscript. We have now received the enclosed reports from the referees. As you will see, both referees 1 and 3 ask for some more data and in the cross-comments all referees agree that these data should be provided. Please co-submit a point-by-point response to all comments with your final ms, and please let me know if you have any questions or comments.

A few editorial requests will also need to be addressed before we can proceed with the official acceptance of your manuscript:

- With the 5 main figures, we will publish your ms as a short report. The results section needs to be called "Results and Discussion".

- The subheading "Data Availability Section" needs to be added.

- The conflict of interest subheading needs to be "Disclosure and Competing Interests Statement"

- There is one author name discrepancy: Chantelle Sephton (in our online system) vs Chantelle P. Sephton (in the ms). Please correct.

- The FUNDING INFO needs to be congruent in our online system and in the ms file. Grant numbers missing in manuscript: Natural Sciences and Engineering Research Council of Canada (NSERC) - RGPIN-2018-06227, DGECR-2018-00093; Brain Canada Foundation; Canadian Institutes of Health Research (CIHR)- 202409PJT-526975;Canadian Institutes of Health Research (CIHR) - 202309PJT-506236;Natural Sciences and Engineering Research Council of Canada (NSERC)- RGPIN-2020-06376;Natural Sciences and Engineering Research Council of Canada (NSERC)- DGECR-2020-00060;Canadian Institutes of Health Research (CIHR) IC135391.

- A callout is missing for Figure 5E. The figure 5 legend lists F instead of E.

- The movie needs to be zipped up with its legend provided in a text file; and needs to be labeled as Movie EV1.

- Table EV1 and EV2 should be part of the Reagents and Tools table. The Materials and Methods section should include a separate Reagents and Tools Table file (listing key reagents, experimental models, software and relevant equipment and including their sources and relevant identifiers) and a Methods and Protocols section in which methods should be described using a step-by-step protocol format with bullet points. More information and a template for the Reagents and Tools table is available in our guide to authors online: https://link.springer.com/journal/44319/submission-guidelines

- The ms section order needs to be corrected: It should be title page with complete author information, abstract, introduction, results, discussion, methods, data availability section, acknowledgements, disclosure and competing interests statement, references, main figure legends, tables, expanded view figure legends.

- During our routine image/figure checks, we noticed that the microscopy panels across the figure set appear pixelated. This is a common result of converting original 16-bit TIFF images to RGB jpeg format for publication, and while not a cause for concern, it can sometimes give the impression of image alteration to critical readers.
To resolve this, please upload the microscopy source data for the entire figure set using the original captured 16-Bit image. You can also upload this source data on the BioStudies website. Currently, the source data is pixelated in RGP JPEG format. This will allow us to confirm the integrity of the full figure set and support transparency for readers.

* Figure Legends - Comments *

- Please note that the exact p values are not provided in the legends of figures 1B, D, J, K; 3D, E, F, G, H, I; 4A-D; 5A-D; EV1 D-F; EV2 A, C, D, E; EV3 D, EV4 A, B; EV5 B-F. Please provide exact values as reasonable.
- Please note that information related to n is missing in the legends of figures 1B, E

EMBO press papers are accompanied online by A) a short (1-2 sentences) summary of the findings and their significance, B) 2-3 bullet points highlighting key results and C) a synopsis image that is exactly 550 pixels wide and 200-600 pixels high (the height is variable). The synopsis image should provide a sketch of the major findings, like a graphical abstract. Please note that text needs to be readable at the final size. Please send us this information along with the final manuscript.

I look forward to seeing a final version of your manuscript as soon as possible.

Best regards,
Esther

Esther Schnapp, PhD
Senior Editor
EMBO reports

Referee #1:

The authors have generated new dadaa and adequately responded to my points 2,5 and 6. For the remaining points (1,3,4) the n number is simply not convincing for the results reported. The justification of high resolution tools and methods (e.g. Licor or MitoSox) cannot justify an n number of 2-3. Moreover the claims made by the authors for statistical significance are not valid given the small number of biological replicates.

Referee #2:

The authors have added additional data showing that rapamycin or NAC treatment reverts several of the phenotypes of GATOR1-deficient astrocytes. These data provide direct evidence that increased mTORC1 activity and ROS generation lead to the metabolic defects observed upon loss of GATOR1. This is relevant study that will be of interest to the readership of EMBO reports.

Referee #3:

Thank you to the authors for the quick turnaround in their manuscript and for addressing most of my comments.

I think to really strengthen the CII/ mitochondrial metabolism angle of the paper, it would have been prudent to do the oroborus experiments (Fig.3) with rapamycin/ rescue experiments and the blue native PAGE. I think this is the exciting and novel part of the project.

Cross-comments from referee 1:

Regarding my comments on the revisions, I would like to stand by them, as I would not feel confident to endorse a study with low n numbers. Moreover, the explanation provided (use of high resolution methodologies) is not sufficient in my opinion. I also agree with the comment by Reviewer #3 and I believe the authors should address that too.

Cross-comments from referee 3:

I am inclined to agree with Reviewer 1.

I understand the authors argument that this is a short report and will be a springboard for follow-up studies (which I think will be very interesting), but in my opinion that means the central claim of the report needs to be very robust to be of interest to the general audience of EMBO Reports.

Cross-comments from referee 2:

I don't think that the authors have to extend their investigations, but data robustness of course is vital. If the other referee's voice concerns in this regard, I agree that this should be taken under consideration.

Referee #1:

The authors have generated new dadaa and adequately responded to my points 2,5 and 6. For the remaining points (1,3,4) the n number is simply not convincing for the results reported. The justification of high resolution tools and methods (e.g. Licor or MitoSox) cannot justify an n number of 2-3. Moreover the claims made by the authors for statistical significance are not valid given the small number of biological replicates.

We thank the reviewer for their careful evaluation of the figures and statistical concern. We acknowledge your concern of using n=3. We have processed additional cohorts of the mice, boosting the individual mice analyzed in these assays to 5-8 per genotype. While this has not changed our interpretation, it has strengthened the statistics and given us additional confidence in our observations. We have also revised all figure legends to improve clarity, explicitly define biological replication, indicate the exact p-values within the figure panels, and ensure full compliance with EMBO Reports reporting standards. Specifically, we have:

A) Clarified biological replication ("n"). Across all figures and EV figures, we have explicitly defined *n* as the number of independent biological replicates, specifying whether these correspond to individual mice, independent cell cultures, or independent biological experiments, clearly. Technical sampling (multiple imaging fields per culture) is also clearly distinguished from biological replication. For western blot analysis, we now explicitly state when a blot is representative of other replicates, and clarify that densitometry quantification includes all biological replicates (i.e. representative blots + others from independent experiments). We believe this resolves any ambiguity between the number of lanes displayed and the number of samples quantified.

B) In accordance with EMBO Reports requirements, exact P-values have been added to the legends within reason, or integrated into the panels as flagged by the reviewer and editor.

C) For normalization of the phospho-protein analysis, figure legends now explicitly describe the normalization strategy (P-protein normalized to corresponding total protein and then to the loading control, b-actin), ensuring transparency and consistency across figures.

We note that the conclusions of the manuscript do not rely on any single assay, but are supported by convergent evidence from multiple independent approaches, including biochemical analysis, imaging, metabolomics, respirometry, gene expression, and *in vivo* phenotying. This cross-validation substantially strengthens

the confidence in our observations and interpretation.

Referee #2:

The authors have added additional data showing that rapamycin or NAC treatment reverts several of the phenotypes of GATOR1-deficient astrocytes. These data provide direct evidence that increased mTORC1 activity and ROS generation lead to the metabolic defects observed upon loss of GATOR1. This is relevant study that will be of interest to the readership of EMBO reports.

We thank the reviewer for their constructive analysis and their positive assessment of the study. We are pleased that the added pharmacological rescue experiments have strengthened the mechanistic framework linking mTORC1 activity, redox imbalance, and metabolic dysfunction downstream of astrocytic GATOR1 deficiency.

Referee #3:

Thank you to the authors for the quick turnaround in their manuscript and for addressing most of my comments.

I think to really strengthen the CII/ mitochondrial metabolism angle of the paper, it would have been prudent to do the oroborus experiments (Fig.3) with rapamycin/ rescue experiments and the blue native PAGE. I think this is the exciting and novel part of the project.

We thank the reviewer for their positive assessment of the manuscript and for highlighting the importance and novelty of the mitochondrial Complex II-linked metabolic phenotype. We agree that a further and future mechanistic dissection of mitochondrial regulation using pharmacological rescue during high-resolution respirometry and blue native PAGE analysis of respiratory complexes are of strong interest and represents an important direction for future work. Indeed, these approaches represent important future directions to further resolve how mTORC1 activity and redox state influence mitochondrial organization and function downstream of GATOR1. The CII-associated respiration and redox phenotypes reported here are supported by multiple independent and convergent datasets, including high-resolution respirometry, metabolomics, redox measurements, gene expression analysis, and *in vivo* validation. Together, we believe these data strongly support the central mechanistic conclusions of this manuscript.

Cross-comments from referee 1:

Regarding my comments on the revisions, I would like to stand by them, as I would not feel confident to endorse a study with low n numbers. Moreover, the explanation provided (use of high resolution methodologies) is not sufficient in my opinion. I also agree with the comment by Reviewer #3 and I believe the authors should address that too.

We acknowledge the position regarding biological replication thresholds and we have taken this point seriously. To summarize our direct response above, we have now increased the n-values where appropriate and clearly state that all n-values represent independent biological replicates or independent experimental replicates from independent cultures, and report the exact statistical values, within the figure panels. Where appropriate, statistical tests are now detailed in each figure legend.

We note that the conclusions of the study are not based on individual datasets, but on convergent evidence from multiple independent and orthogonal approaches, including biochemistry, imaging, metabolomics, mitochondrial respirometry, gene expression, and in vivo analyses. We therefore believe that the strength of our data and analysis are appropriate for the mechanistic scope of the work and consistent with current practices in EMBO Reports.

With respect to the comment by Reviewer 3, we have addressed this point where feasible and clarified additional experiments for future directions that extend beyond the aims of the present study.

Cross-comments from referee 3:

I am inclined to agree with Reviewer 1.

I understand the authors argument that this is a short report and will be a springboard for follow-up studies (which I think will be very interesting), but in my opinion that means the central claim of the report needs to be very robust to be of interest to the general audience of EMBO Reports.

We agree and thank the reviewer for their perspective. We believe this criterion is appropriately met, as the core claims are supported by consistent observations across multiple independent and orthogonal approaches, including mitochondrial respirometry, metabolomics, redox measurements, gene expression analyses, and in

vivo validation. These data now include addition biological replicates, which have further strengthened the statistical analysis and confidence in our interpretation.

As previously, the central conclusions do not rely on a single assay or experimental modality, but instead emerge from the convergence of these datasets, which together establish a coherent mechanistic framework linking astrocytic GATOR1 loss to mitochondrial Complex II–associated metabolic and redox dysfunction. The data is robust, statistically analyzed, and self-contained for the intended audience, while also motivating for follow-up studies extending beyond this manuscript.

Cross-comments from referee 2:

I don't think that the authors have to extend their investigations, but data robustness of course is vital. If the other referee's voice concerns in this regard, I agree that this should be taken under consideration.

We agree that data robustness is essential. In response to this concern, we have increased the number of biological replicates in the experiments where statistical power was limited. The expanded datasets strengthen the statistical analysis while preserving the direction and magnitude of the reported effects. Importantly, the central findings are consistently supported across multiple independent and orthogonal experimental approaches, including primary astrocyte cultures and histological analyses. All revised figures now clearly indicate biological replication and exact statistical values. We believe these additions substantially strengthen the robustness and clarity of the study.

Referee #1:

Comments to authors:

I am disappointed to see that instead of responding with either increased numbers of experiments or some clear information on statistics, the authors have opted for a response that aims to quiet reviewer concerns.

In response to my comments which had 2 overarching points: a) increase n number and b) report statistics transparently, the authors have expanded the respective methods section.

I am unsure about the following statements in the author's response and in the stats section:
"...standard practice in mechanistic biological research and EMBO Reports."

"Prior to application of parametric tests, datasets were visually inspected for distribution symmetry and evaluated for comparable variance between groups to ensure suitability of model selection."

1) Standard practice for all journals is transparent and mathematically correct statistical analysis. There are no shortcuts.

2) Distribution symmetry or normality: The standard tests include Shapiro-Wilk, Anderson-Darling, D'Agostino-Pearson, and sometimes Kolmogorov-Smirnov/Lilliefors. For Variance: Bartlettt's and Levene's test.

These need to be in a table with numbers and clear results to make any claims about symmetry/distribution and variance and to correctly select downstream models.
Alternatively: For a two-group mean comparison, instead of doing test normality->test equal variance->choose t-test/Welch/Mann–Whitney, authors could straight use Welch's t-test by default because Welch's test does not require equal variances and usually performs well when variances differ.

Importantly, visual inspection is subject to bias. Could the authors explain how the following figure panels were categorized after visual inspection in terms of normality and variance? E.g. Fig. 3I SOD signal (4 control, 8 KO), Fig. 4A,B, D Mitosox (not clear from legend regarding n numbers), Fig. 5 B, D, Fig. EV1 E, F multiple panels and more. No answer is required if formal mathematical tests are run and reported, which will justify the selection of statistical test and remove the bias of eyeballing data symmetry and variance.

I think reviewer 1 makes some very valid concerns and I would want to see some things changed to make this appropriate for publication. Ultimately this doesn't feel like it needs major revisions, but in my opinion it does need:

- Some tests changed
- Some tests more clearly described
- Consistent statistical reporting on graphs i.e. either exact values or cut offs (realistically it should be the former)
- Publication of the data which went into each graph so people can run their own analyses/remake the graphs OR a set of statistical reporting of the outputs of the tests they run.

For more detail I have gone through the reviewer comments below. To try and make it as clear as possible I figured I would structure my reply as almost a response to reviewers:

Comments to authors:
I am disappointed to see that instead of responding with either increased numbers of experiments or some clear information on statistics, the authors have opted for a response that aims to quiet reviewer concerns.
In response to my comments which had 2 overarching points: a) increase n number and b) report statistics transparently, the authors have expanded the respective methods section.
I am unsure about the following statements in the author's response and in the stats section:
"...standard practice in mechanistic biological research and EMBO Reports."
"Prior to application of parametric tests, datasets were visually inspected for distribution symmetry and evaluated for comparable variance between groups to ensure suitability of model selection."
This is not acceptable practice, I agree with reviewer 1 that it is a very minor request that they do at least test their data for normality if they are going to do tests which assume that distribution. There are limits to this, the sample sizes are low so and so it would take quite a strong deviation from normality to flag something up in the tests but that is more appropriate than the visual inspection they describe which is entirely subjective.. As such I think they should, minimum, perform one of these tests, and then to report that this was carried out preferably in the figure legend along with the other tests being used.
Standard practice for all journals is transparent and mathematically correct statistical analysis. There are no shortcuts.
Distribution symmetry or normality: The standard tests include Shapiro-Wilk, Anderson-Darling, D'Agostino-Pearson, and sometimes Kolmogorov-Smirnov/Lilliefors. For Variance: Bartlettt's and Levene's test.
These need to be in a table with numbers and clear results to make any claims about symmetry/distribution and variance and to correctly select downstream models.
I do think that this would be ideal to have such a table, however, what would be even better would be to have the data available in an associated excel sheet. Then anyone can replot the graphs and do their own statistical analyses which is even more transparent.

Alternatively: For a two-group mean comparison, instead of doing test normality->test equal variance->choose t-test/Welch/Mann-Whitney, authors could straight use Welch's t-test by default because Welch's test does not require equal variances and usually performs well when variances differ.
This is also a fair point, it is not a huge concession to do Welch's t-test to not assume equal variance. I guess the difficulty comes if their data is not normally distributed as for some of these analyses this would make them underpowered to detect a change i.e. it is impossible with n=3 to get p<0.05 by Mann-Whitney. However, if they are not normally distributed then that is something they would need to address by increasing n.
Importantly, visual inspection is subject to bias. Could the authors explain how the following figure panels were categorized after visual inspection in terms of normality and variance? E.g. Fig. 3I SOD signal (4 control, 8 KO), Fig. 4A,B, D Mitosox (not clear from legend regarding n numbers), Fig. 5 B, D, Fig. EV1 E, F multiple panels and more. No answer is required if formal mathematical tests are run and reported, which will justify the selection of statistical test and remove the bias of eyeballing data symmetry and variance.
I guess it is informative to think about a few examples, for why I think this reviewer is correct on these points (and to raise some other concerns I only pick up 1 instance of each issue but they persist):

Figure 1K – I'm unsure how they think those 2 groups have equal variance since 1 group seems to have 0 variance. It looks a lot like one group has been used to normalise the other meaning that every value in 1 group is equal to 1A.U. If this is the case then a 2 sample t-test is entirely inappropriate and instead they should be doing a 1 sample t-test to test whether the KO value is significantly different to 1A.U.

Figure 2H – it is not reported if they corrected for multiple testing. I understand the temptation to treat each thing as individual, however, this is metabolomic data making it similar to, say, RNAseq where in the absence of multiple testing correction we have various issues.

Figure 3B – They report using a one way ANOVA, but they have error bars between specific groups. This means they have used a post-hoc test (perfectly fine) however, they haven't reported what that is in the legend. This also means they haven't reported if they correct for multiple testing again.

Personal preference:

Throughout I am unsure why they sometimes report exact p-values (although always with p less than or equal to e.g. Fig 3E p=< 0.023... I would assume this is p=0.023 so maybe just report the exact p value?), at other times they show standard cut offs e.g. p<0.05 or p<0.01, and then they use n.s. repeatedly. I think they should pick a convention, my preference would be always to report the exact p values as a p=0.051 not significant value is not really different to a p<0.05 which is p=0.049.

Figure 1G & 1H & 3B & 3C etc. – I would be tempted to remove the statistical test entirely since with n=3 absence of evidence is not evidence of absence and I think these tests are relatively meaningless, anyone can look at this and observe there is no apparent difference.

Response to Statistical Advisor:

We thank the statistical advisor for the clear and constructive suggestions to improve our manuscript. Please see our response below:

This is not acceptable practice, I agree with reviewer 1 that it is a very minor request that they do at least test their data for normality if they are going to do tests which assume that distribution. There are limits to this, the sample sizes are low so and so it would take quite a strong deviation from normality to flag something up in the tests but that is more appropriate than the visual inspection they describe which is entirely subjective.. As such I think they should, minimum, perform one of these tests, and then to report that this was carried out preferably in the figure legend along with the other tests being used.

1. We thank the advisor for this clarification. We agree that formal assessment of model assumptions is preferable to descriptive language. We have therefore removed the phrasing referring to "visual inspection" and revised the Statistical Analysis section to specify the criteria used for test selection.

For two-group comparisons, we applied the recommended Welch's *t*-test, which does not assume equal variances and is robust for heteroscedasticity, thereby removing the need for formal equal-variance testing. For immunohistochemical (IHC) quantifications, staining and imaging were performed in independent biological experiments, and quantifications were grouped accordingly. Within each independent experiment, the mean WT signal was normalized to 1, and corresponding KO values were expressed relative to their matched WT control. Statistical testing was then performed across independent biological replicates (experiments), treating each experiment as the statistical unit. WT normalization removed variance n the control group and a one-sample test against 1 A.U. was applied, as described. For experiments involving more than two-groups, we have explicitly reported use of one-way ANOVA followed by Tukey's post-hoc test.

I do think that this would be ideal to have such a table, however, what would be even better would be to have the data available in an associated excel sheet. Then anyone can replot the graphs and do their own statistical analyses which is even more transparent.

2. Exact p-values are now consistently reported, and the underlying numerical datasets have been consolidated into Dataset EV1 to allow independent verification and reanalysis.

This is also a fair point, it is not a huge concession to do Welch's t-test to not assume equal variance. I guess the difficulty comes if their data is not normally distributed as for some of these analyses this would make them underpowered to detect a change i.e. it is impossible with n=3 to get p<0.05 by Mann-Whitney. However, if they are not normally distributed then that is something they would need to address by increasing n.

3. As indicated above, we applied the recommended Welch's *t*-tests.

Figure 1K – I'm unsure how they think those 2 groups have equal variance since 1 group seems to have 0 variance. It looks a lot like one group has been used to normalise the other meaning that every value in 1 group is equal to 1A.U. If this is the case then a 2 sample t-test is entirely inappropriate and instead they should be doing a 1 sample t-test to test whether the KO value is significantly different to 1A.U.

4. We appreciate the clarification regarding panels such as Fig. 1K. In these experiments, values were normalized within each independent biological replicate such that the mean WT signal from each experiment was set to 1, and corresponding KO samples were expressed relative to that matched control. This normalization procedure removes variance in the WT group by design. We agree that under these conditions a two-sample comparison is not appropriate and have therefore reanalyzed the KO values using a one-sample test against the normalized reference value of 1 A.U., treating each independent experiment as the statistical unit. The corresponding p-value has been updated in the revised figure and legend.

Figure 2H – it is not reported if they corrected for multiple testing. I understand the temptation to treat each thing as individual, however, this is metabolomic data making it similar to, say, RNAseq where in the absence of multiple testing correction we have various issues.

We appreciate the advisor's point regarding metabolomics. These experiments were conducted using a targeted mass-spectrometry approach focused on a predefined panel of biologically selected metabolites relevant to mitochondrial metabolism. These analyses were hypothesis-driven and the number of metabolites examined were limited accordingly. Each metabolite extract condition was normalized to total protein content prior to statistical analysis. Because this approach differs fundamentally from high-dimensional untargeted –omics datasets (eg. RNAseq), each metabolite was evaluated individually using the specified statistical tests. We thank the statistical advisor for raising this distinction and clarify this point in the revised manuscript.

Figure 3B – They report using a one way ANOVA, but they have error bars between specific groups. This means they have used a post-hoc test (perfectly fine) however, they haven't reported what that is in the legend. This also means they haven't reported if they correct for multiple testing again.

We have now clarified in the legends, where appropriate, that ANOVA with post-hoc Tukey was used.

Throughout I am unsure why they sometimes report exact p-values (although always with p less than or equal to e.g. Fig 3E p=< 0.023... I would assume this is p=0.023 so maybe just report the exact p value?), at other times they show standard cut offs e.g. p<0.05 or p<0.01, and then they use n.s. repeatedly. I think they should pick a convention, my preference would be always to report the exact p values as a p=0.051 not significant value is not really different to a p<0.05 which is p=0.049.

Regarding reporting conventions, we have adopted a consistent format of reporting exact p-values to 3-decimal places where applicable.

Figure 1G & 1H & 3B & 3C etc. – I would be tempted to remove the statistical test entirely since with n=3 absence of evidence is not evidence of absence and I think these

tests are relatively meaningless, anyone can look at this and observe there is no apparent difference.

Regarding Figure 1G, 1H, etc. where 3 independent measurements were used for statistical testing, we have removed the statistical tests entirely, as suggested, to avoid over-interpretation.

Dr. Paul Dutchak
Laval University
Psychiatry and Neuroscience
Canada

Dear Paul,

I am pleased to accept your manuscript for publication in the next available issue of EMBO reports. Thank you for your contribution to our journal.

Your manuscript will be processed for publication by EMBO Press. It will be copy edited and you will receive page proofs prior to publication. Please note that you will be contacted by Springer Nature Author Services to complete licensing and payment information.

You may qualify for financial assistance for your publication charges - either via a Springer Nature fully open access agreement or an EMBO initiative. Check your eligibility: https://link.springer.com/journal/44319/how-to-publish-with-us

Should you be planning a Press Release on your article, please notify the production team via this form in order to coordinate publication and release dates: https://forms.cloud.microsoft/e/eH4A8JVP6g

If you have any questions, please do not hesitate to contact the Editorial Office. Thank you for your contribution to EMBO Reports.

Best regards,
Esther

Esther Schnapp, PhD
Senior Editor
EMBO reports

>>> Please note that it is EMBO Reports policy for the transcript of the editorial process (containing referee reports and your response letter) to be published as an online supplement to each paper. If you do NOT want this, you will need to inform the Editorial Office via email immediately. More information is available here: https://link.springer.com/partners/embo-press/editorial-policies#Peer%20review

EMBO Press Author Checklist

Corresponding Author Name: Paul Dutchak
Journal Submitted to: EMBO Reports
Manuscript Number: EMBOR-2025-62872V1

USEFUL LINKS FOR COMPLETING THIS FORM
The EMBO Journal - Author Guidelines
EMBO Reports - Author Guidelines
Molecular Systems Biology - Author Guidelines
EMBO Molecular Medicine - Author Guidelines

Reporting Checklist for Life Science Articles (updated January
This checklist is adapted from Materials Design Analysis Reporting (MDAR) Checklist for Authors. MDAR establishes a minimum set of requirements in transparent reporting in the life sciences (see Statement of Task: 10.31222/osf.io/9sm4x). Please follow the journal's guidelines in preparing your
Please note that a copy of this checklist will be published alongside your article.

Abridged guidelines for figures
1. Data
The data shown in figures should satisfy the following conditions:
- the data were obtained and processed according to the field's best practice and are presented to reflect the results of the experiments in an accurate and unbiased manner.
- ideally, figure panels should include only measurements that are directly comparable to each other and obtained with the same assay.
- plots include clearly labeled error bars for independent experiments and sample sizes. Unless justified, error bars should not be shown for technical
- if n<5, the individual data points from each experiment should be plotted. Any statistical test employed should be justified.
- Source Data should be included to report the data underlying figures according to the guidelines set out in the authorship guidelines on Data

2. Captions
Each figure caption should contain the following information, for each panel where they are relevant:
- a specification of the experimental system investigated (eg cell line, species name).
- the assay(s) and method(s) used to carry out the reported observations and measurements.
- an explicit mention of the biological and chemical entity(ies) that are being measured.
- an explicit mention of the biological and chemical entity(ies) that are altered/varied/perturbed in a controlled manner.
- the exact sample size (n) for each experimental group/condition, given as a number, not a range;
- a description of the sample collection allowing the reader to understand whether the samples represent technical or biological replicates (including how many animals, litters, cultures, etc.).
- a statement of how many times the experiment shown was independently replicated in the laboratory.
- definitions of statistical methods and measures:
  - common tests, such as t-test (please specify whether paired vs. unpaired), simple χ2 tests, Wilcoxon and Mann-Whitney tests, can be unambiguously identified by name only, but more complex techniques should be described in the methods section;
  - are tests one-sided or two-sided?
  - are there adjustments for multiple comparisons?
  - exact statistical test results, e.g., P values = x but not P values < x;
  - definition of 'center values' as median or average;
  - definition of error bars as s.d. or s.e.m.

Please complete ALL of the questions below.
Select "Not Applicable" only when the requested information is not relevant for your study.

Materials

Newly Created Materials	Information included in the manuscript?	In which section is the information available? (Reagents and Tools Table, Materials and Methods, Figures, Data Availability Section)
New materials and reagents need to be available; do any restrictions apply?	Not Applicable	

Antibodies	Information included in the manuscript?	In which section is the information available? (Reagents and Tools Table, Materials and Methods, Figures, Data Availability Section)
For **antibodies** provide the following information:<br>- Commercial antibodies: RRID (if possible) or supplier name, catalogue number and or/clone number<br>- Non-commercial: RRID or citation	Yes	Table EV2

DNA and RNA sequences	Information included in the manuscript?	In which section is the information available? (Reagents and Tools Table, Materials and Methods, Figures, Data Availability Section)
**Short novel DNA or RNA including primers, probes**: provide the sequences.	Yes	Table EV1

Cell materials	Information included in the manuscript?	In which section is the information available? (Reagents and Tools Table, Materials and Methods, Figures, Data Availability Section)
**Cell lines:** Provide species information, strain. Provide accession number in repository **OR** supplier name, catalog number, clone number, and/**OR** RRID.	Yes	Materials and Methods
**Primary cultures:** Provide species, strain, sex of origin, genetic modification status.	Yes	Materials and Methods
Report if the cell lines were recently **authenticated** (e.g., by STR profiling) and tested for mycoplasma contamination.	Not Applicable	

Experimental animals	Information included in the manuscript?	In which section is the information available? (Reagents and Tools Table, Materials and Methods, Figures, Data Availability Section)
**Laboratory animals or Model organisms:** Provide species, strain, sex, age, genetic modification status. Provide accession number in repository **OR** supplier name, catalog number, clone number, **OR** RRID.	Yes	Materials and Methods
**Animal observed in or captured from the field:** Provide species, sex, and age where possible.	Yes	Materials and Methods and Supplemental Video
Please detail **housing and husbandry conditions**.	Not Applicable	

Plants and microbes	Information included in the manuscript?	In which section is the information available? (Reagents and Tools Table, Materials and Methods, Figures, Data Availability Section)
**Plants:** provide species and strain, ecotype and cultivar where relevant, unique accession number if available, and source (including location for collected wild specimens).	Not Applicable	
**Microbes:** provide species and strain, unique accession number if available, and source.	Not Applicable	

Human research participants	Information included in the manuscript?	In which section is the information available? (Reagents and Tools Table, Materials and Methods, Figures, Data Availability Section)
If collected and within the bounds of privacy constraints report on age, sex and gender or ethnicity for all study participants.	Not Applicable	

Core facilities	Information included in the manuscript?	In which section is the information available? (Reagents and Tools Table, Materials and Methods, Figures, Data Availability Section)
If your work benefited from core facilities, was their service mentioned in the acknowledgments section?	Yes	Materials and Methods and Acknowledgments

Design

Study protocol	Information included in the manuscript?	In which section is the information available? (Reagents and Tools Table, Materials and Methods, Figures, Data Availability Section)
If study protocol has been **pre-registered, provide DOI in the manuscript**. For clinical trials, provide the trial registration number **OR** cite DOI.	Not Applicable	
Report the **clinical trial registration number** (at ClinicalTrials.gov or equivalent), where applicable.	Not Applicable	

Laboratory protocol	Information included in the manuscript?	In which section is the information available? (Reagents and Tools Table, Materials and Methods, Figures, Data Availability Section)
Provide DOI OR other citation details if **external detailed step-by-step protocols** are available.	Yes	Materials and Methods

Experimental study design and statistics	Information included in the manuscript?	In which section is the information available? (Reagents and Tools Table, Materials and Methods, Figures, Data Availability Section)
Include a statement about **sample size** estimate even if no statistical methods were used.	Yes	Legends
Were any steps taken to minimize the effects of subjective bias when allocating animals/samples to treatment (e.g. **randomization procedure**)? If yes, have they been described?	Not Applicable	
Include a statement about **blinding** even if no blinding was done.	Yes	Materials and Methods
Describe **inclusion/exclusion criteria** if samples or animals were excluded from the analysis. Were the criteria pre-established?<br><br>If sample or data points were omitted from analysis, report if this was due to attrition or intentional exclusion and provide justification.	Not Applicable	
For every figure, are **statistical tests** justified as appropriate? Do the data meet the assumptions of the tests (e.g., normal distribution)? Describe any methods used to assess it. Is there an estimate of variation within each group of data? Is the variance similar between the groups that are being statistically compared?	Yes	Materials and Methods and Legends

Sample definition and in-laboratory replication	Information included in the manuscript?	In which section is the information available? (Reagents and Tools Table, Materials and Methods, Figures, Data Availability Section)
In the figure legends: state number of times the experiment was **replicated** in laboratory.	Yes	Legends
In the figure legends: define whether data describe **technical or biological replicates**.	Yes	Legends

Ethics

Ethics	Information included in the manuscript?	In which section is the information available? (Reagents and Tools Table, Materials and Methods, Figures, Data Availability Section)
Studies involving **human participants**: State details of **authority granting ethics approval** (IRB or equivalent committee(s), provide reference number for approval.	Not Applicable	
Studies involving **human participants**: Include a statement confirming that **informed consent** was obtained from all subjects and that the experiments conformed to the principles set out in the WMA Declaration of Helsinki and the Department of Health and Human Services Belmont Report.	Not Applicable	
Studies involving **human participants:** For publication of **patient photos**, include a statement confirming that consent to publish was obtained.	Not Applicable	
Studies involving experimental **animals**: State details of **authority granting ethics approval** (IRB or equivalent committee(s), provide reference number for approval. Include a statement of compliance with ethical regulations.	Yes	Materials and Methods
Studies involving **specimen and field samples:** State if relevant **permits** obtained, provide details of authority approving study; if none were required, explain why.	Not Applicable	

Dual Use Research of Concern (DURC)	Information included in the manuscript?	In which section is the information available? (Reagents and Tools Table, Materials and Methods, Figures, Data Availability Section)
Could your study fall under dual use research restrictions? Please check biosecurity documents and list of **select agents and toxins** (CDC): https://www.selectagents.gov/sat/list.htm	Not Applicable	
If you used a select agent, is the security level of the lab appropriate and reported in the manuscript?	Not Applicable	
If a study is subject to dual use research of concern regulations, is the name of the **authority granting approval and reference number** for the regulatory approval provided in the manuscript?	Not Applicable	

Reporting

The MDAR framework recommends adoption of discipline-specific guidelines, established and endorsed through community initiatives. Journals have their own policy about requiring specific guidelines and recommendations to complement MDAR.

Adherence to community standards	Information included in the manuscript?	In which section is the information available? (Reagents and Tools Table, Materials and Methods, Figures, Data Availability Section)
State if relevant guidelines or checklists (e.g., **ICMJE, MIBBI, ARRIVE, PRISMA**) have been followed or provided.	Not Applicable	
For **tumor marker prognostic studies,** we recommend that you follow the **REMARK** reporting guidelines (see link list at top right). See author guidelines, under 'Reporting Guidelines'. Please confirm you have followed these guidelines.	Not Applicable	
For **phase II and III randomized controlled trials**, please refer to the **CONSORT** flow diagram (see link list at top right) and submit the CONSORT checklist (see link list at top right) with your submission. See author guidelines, under 'Reporting Guidelines'. Please confirm you have submitted this list.	Not Applicable	

Data Availability

Data availability	Information included in the manuscript?	In which section is the information available? (Reagents and Tools Table, Materials and Methods, Figures, Data Availability Section)
Have **primary datasets** been deposited according to the journal's guidelines (see 'Data Deposition' section) and the respective accession numbers provided in the Data Availability Section?	Yes	Data Availability section
Were **human clinical and genomic datasets** deposited in a public access-controlled repository in accordance to ethical obligations to the patients and to the applicable consent agreement?	Not Applicable	
Are **computational models** that are central and integral to a study available without restrictions in a machine-readable form? Were the relevant accession numbers or links provided?	Not Applicable	
If publicly available data were reused, provide the respective **data citations in the reference list**.	Not Applicable	
