# Supplementary material for: GATOR1 signaling defects promote astrocytic metabolic rewiring and excitatory neurotransmitter cycling
Source: EMBO Rep. 2026 Jul 3;27(15):4252–71. doi: 10.1038/s44319-026-00846-w (PMC13458053; doi:10.1038/s44319-026-00846-w)
Supplement: Supplementary file 5 [file 44319_2026_846_MOESM5_ESM.zip › Figure 2/Figure 2.I/Glycolysis and PP and TCA cycle.pdf]

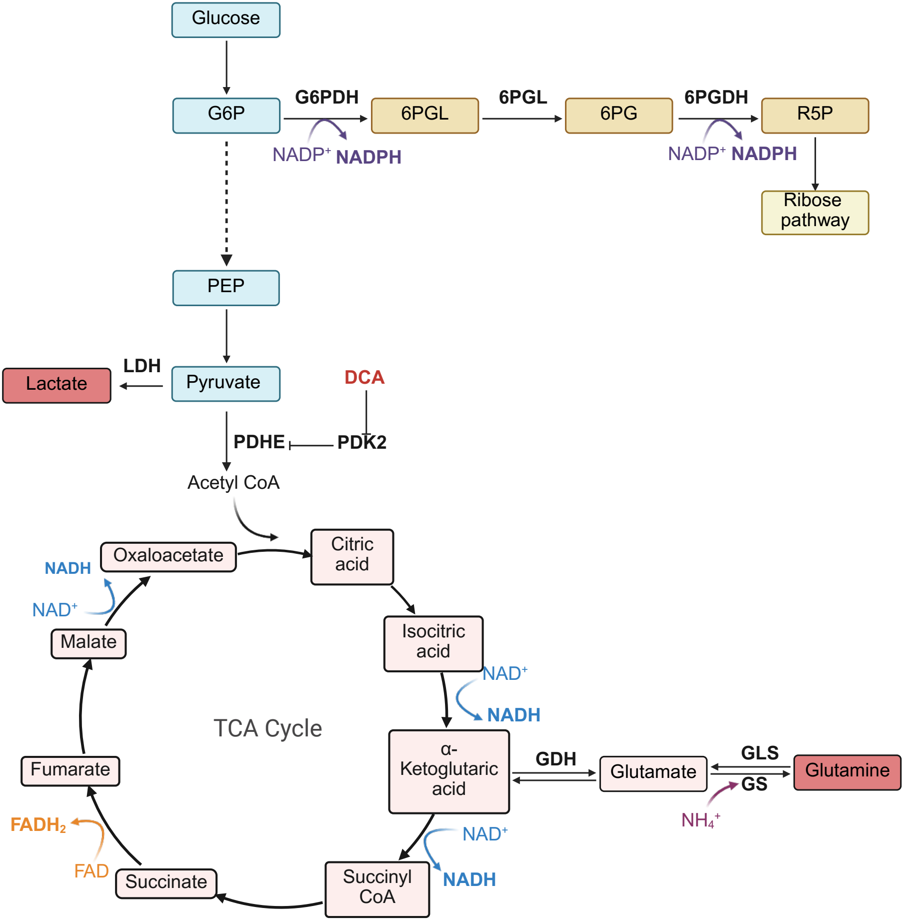
Glucose
G6P
G6PDH
6PGL
6PGL
6PG
6PGDH
R5P
NADP+ NADPH
NADP+ NADPH
Ribose pathway
PEP
LDH
Lactate
Pyruvate
DCA
PDHE
PDK2
Acetyl CoA
Citric acid
Oxaloacetate
NADH
NAD+
Malate
Isocitric acid
NAD+
NADH
TCA Cycle
α-Ketoglutaric acid
GDH
Glutamate
GLS
Glutamine
GS
NH4+
Fumarate
FADH2
FAD
Succinate
Succinyl CoA
NAD+
NADH
