# Supplementary material for: GATOR1 signaling defects promote astrocytic metabolic rewiring and excitatory neurotransmitter cycling
Source: EMBO Rep. 2026 Jul 3;27(15):4252–71. doi: 10.1038/s44319-026-00846-w (PMC13458053; doi:10.1038/s44319-026-00846-w)
Supplement: Supplementary file 8 [file 44319_2026_846_MOESM8_ESM.zip › Figure 5/Figure 5.E/Astrocyte-Neuron schematic.pdf]

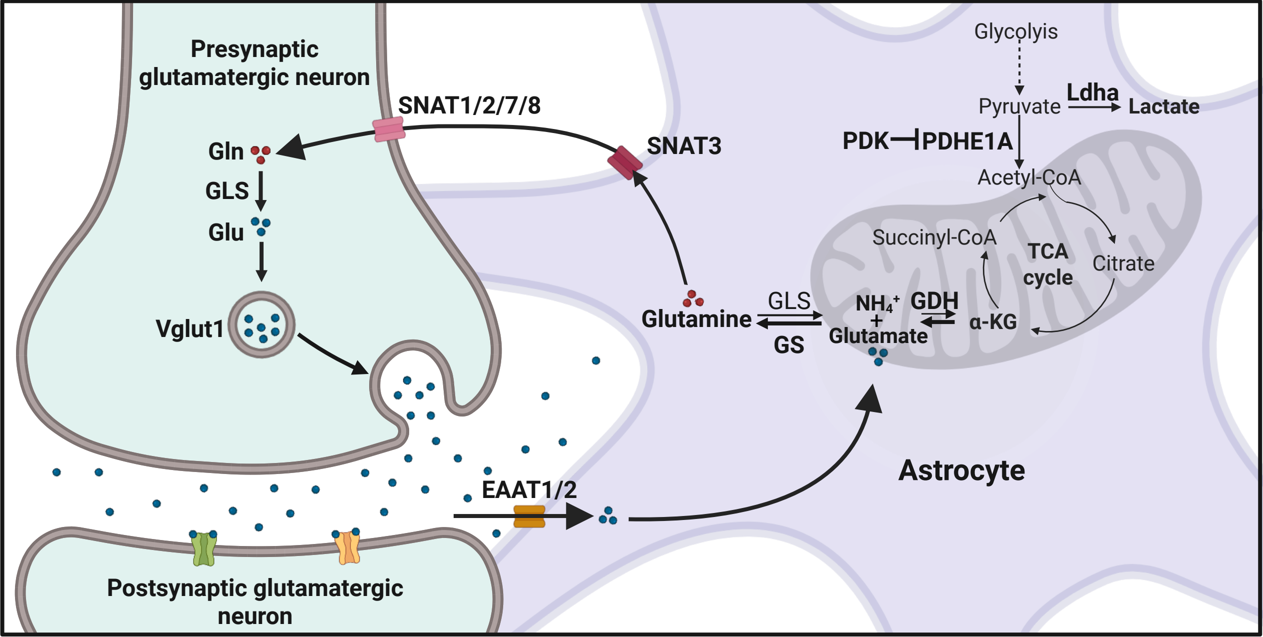

Presynaptic glutamatergic neuron
SNAT1/2/7/8
Gln
GLS
Glu
Vglut1
SNAT3
Glycolyis
Pyruvate
Ldha
Lactate
PDK
PDHE1A
Acetyl-CoA
Succinyl-CoA
TCA cycle
Citrate
Glutamine
GLS
GS
NH_4^+ + Glutamate
GDH
α-KG
Astrocyte
EAAT1/2
Postsynaptic glutamatergic neuron
